# Effect of Colchicine vs Usual Care Alone on Intubation and 28-Day Mortality in Patients Hospitalized With COVID-19: A Randomized Clinical Trial

**DOI:** 10.1001/jamanetworkopen.2021.41328

**Published:** 2021-12-29

**Authors:** Rafael Diaz, Andrés Orlandini, Noelia Castellana, Alberto Caccavo, Pablo Corral, Gonzalo Corral, Carolina Chacón, Pablo Lamelas, Fernando Botto, María Luz Díaz, Juan Manuel Domínguez, Andrea Pascual, Carla Rovito, Agustina Galatte, Franco Scarafia, Omar Sued, Omar Gutierrez, Sanjit S. Jolly, José M. Miró, John Eikelboom, Mark Loeb, Aldo Pietro Maggioni, Deepak L. Bhatt, Salim Yusuf

**Affiliations:** 1 Estudios Clínicos Latino América, Rosario, Argentina; 2 Instituto Cardiovascular de Rosario, Rosario, Argentina; 3 Universidad Nacional de Rosario, Rosario, Argentina; 4 Hospital de Coronel Suárez Raúl Alfredo Caccavo, Universidad Provincial del Sudoeste, Buenos Aires, Argentina; 5 Departamento de Investigación, Facultad de Medicina, Universidad FASTA, Buenos Aires, Argentina; 6 Infectología Clínica de Mayo, Mar del Plata, Argentina; 7 Universidad Abierta Interamericana, Rosario, Argentina; 8 Unidad Coronaria de Sanatorio Delta de Rosario, Rosario, Argentina; 9 Comite de Epidemiologia y Prevención Cardiovasculr de la Federación Argentina de Cardiologia, Rosario, Argentina; 10 Health Research Methods, Evidence, and Impact, Population Health Research Institute, Hamilton Health Sciences, McMaster University, Hamilton, Canada; 11 Instituto Cardiovascular de Buenos Aires, Buenos Aires, Argentina; 12 Heart Failure and Heart Transplant Unit, Instituto Cardiovascular de Rosario, Rosario, Argentina; 13 Statistics Department, Universidad Nacional de Rosario, Rosario, Argentina; 14 Fundación Huésped, Buenos Aires, Argentina; 15 Ministerio de Salud de Jujuy, Jujuy, Argentina; 16 Division of Cardiology, Population Health Research Institute, Hamilton Health Sciences, McMaster University, Hamilton, Canada; 17 Infectious Diseases Service, Hospital Clínic, Instituto de Investigaciones Biomédicas August Pi i Sunyer, University of Barcelona, Barcelona, Spain; 18 Medicine, Population Health Research Institute, Hamilton Health Sciences, McMaster University, Hamilton, Canada; 19 Departments of Pathology and Molecular Medicine, Population Health Research Institute, Hamilton Health Sciences, McMaster University, Hamilton, Canada; 20 Associazione Nazionale Medici Cardiologi Ospedalieri Research Center, Florence, Italy; 21 Brigham and Women’s Hospital and Harvard Medical School, Boston, Massachusetts

## Abstract

**Importance:**

Hospitalized patients with COVID-19 pneumonia have high rates of morbidity and mortality.

**Objective:**

To assess the efficacy of colchicine in hospitalized patients with COVID-19 pneumonia.

**Design, Setting, and Participants:**

The Estudios Clínicos Latino América (ECLA) Population Health Research Institute (PHRI) COLCOVID trial was a multicenter, open-label, randomized clinical trial performed from April 17, 2020, to March 28, 2021, in adults with confirmed or suspected SARS-CoV-2 infection followed for up to 28 days. Participants received colchicine vs usual care if they were hospitalized with COVID-19 symptoms and had severe acute respiratory syndrome or oxygen desaturation. The main exclusion criteria were clear indications or contraindications for colchicine, chronic kidney disease, and negative results on a reverse transcription–polymerase chain reaction test for SARS-CoV-2 before randomization. Data were analyzed from June 20 to July 25, 2021.

**Interventions:**

Patients were assigned in a 1:1 ratio to usual care or usual care plus colchicine. Colchicine was administered orally in a loading dose of 1.5 mg immediately after randomization, followed by 0.5 mg orally within 2 hours of the initial dose and 0.5 mg orally twice a day for 14 days or discharge, whichever occurred first.

**Main Outcomes and Measures:**

The first coprimary outcome was the composite of a new requirement for mechanical ventilation or death evaluated at 28 days. The second coprimary outcome was death at 28 days.

**Results:**

A total of 1279 hospitalized patients (mean [SD] age, 61.8 [14.6] years; 449 [35.1%] women and 830 [64.9%] men) were randomized, including 639 patients in the usual care group and 640 patients in the colchicine group. Corticosteroids were used in 1171 patients (91.5%). The coprimary outcome of mechanical ventilation or 28-day death occurred in 160 patients (25.0%) in the colchicine group and 184 patients (28.8%) in the usual care group (hazard ratio [HR], 0.83; 95% CI, 0.67-1.02; *P* = .08). The second coprimary outcome, 28-day death, occurred in 131 patients (20.5%) in the colchicine group and 142 patients (22.2%) in the usual care group (HR, 0.88; 95% CI, 0.70-1.12). Diarrhea was the most frequent adverse effect of colchicine, reported in 68 patients (11.3%).

**Conclusions and Relevance:**

This randomized clinical trial found that compared with usual care, colchicine did not significantly reduce mechanical ventilation or 28-day mortality in patients hospitalized with COVID-19 pneumonia.

**Trial Registration:**

ClinicalTrials.gov Identifier: NCT04328480

## Key Points

**Question:**

Does colchicine prevent intubation and mortality in hospitalized patients with COVID-19 pneumonia?

**Findings:**

In this randomized clinical trial of 1279 patients hospitalized with COVID-19, patients allocated to receive colchicine plus usual care or to usual care alone demonstrated no significant difference in the coprimary outcome of mechanical ventilation or 28-day mortality.

**Meaning:**

This randomized clinical trial found that colchicine did not significantly reduce the need for mechanical ventilation or 28-day mortality in patients hospitalized with COVID-19 pneumonia.

## Teaser

This randomized clinical trial assesses the efficacy of colchicine in preventing mechanical ventilation and mortality in hospitalized patients with COVID-19 pneumonia.

## Introduction

Respiratory complications leading to intubation or death occur in 25% of patients admitted to the hospital with COVID-19.^1^ Dexamethasone^2^ and tocilizumab^3^ have reduced mortality in hospitalized patients with severe COVID-19 pneumonia, whereas hydroxychloroquine, chloroquine, interferon, remdesivir, azithromycin, and convalescent plasma^4,5,6,7^ failed to do so.

While the respiratory failure, severe lung involvement, and multiple system failure described in the most severe cases of COVID-19 are not fully understood, the increase in inflammatory markers indicates cytokine release is contributing to increased morbidity and mortality.^8^ The potentially beneficial effect of immunosuppressive agents validates this hypothesis.

Colchicine, an immunomodulatory and anti-inflammatory agent, may have beneficial effects on COVID-19–mediated hyperactivated inflammatory response through the inhibition of tubulin polymerization, cellular adhesion molecules and inflammatory chemokines, inhibition of the activated inflammasome, monocyte migration, secretion of matrix metalloproteinase, and modulation of the pro-thrombotic state.^8^ Colchicine, commonly used for the treatment or prevention of gout and familial Mediterranean fever, has also been shown to reduce cardiovascular events in individuals with atherosclerosis when used at low doses.^9,10^ It has mild side effects and is available at low cost in most countries. Therefore, we developed the Estudios Clínicos Latino América (ECLA)–Population Health Research Institute (PHRI) COLCOVID trial in March 2020, a trial testing colchicine vs usual care in hospitalized patients with a suspected diagnosis of COVID-19 pneumonia.^11,12^

## Methods

This randomized clinical trial was designed and carried out in full adherence to the principles of the Declaration of Helsinki^13^ as well as the laws and regulations of Argentina that fully adhered to the principles outlined in the International Ethical Guidelines for Health-Related Research with Human Beings prepared by the Council of International Organizations of Medical Sciences in collaboration with the World Health Organization. The applicable regulatory bodies approved the trial protocol, presented in Supplement 1, and informed consent forms for each jurisdiction where participating sites were located. Three methods of obtaining informed consent were accepted by our local health authorities (eMethods in Supplement 2). All participants provided informed consent. This study followed the Consolidated Standards of Reporting Trials (CONSORT) reporting guideline.

### Trial Design

The ECLA PHRI COLCOVID Trial was an open-label, multicenter, randomized clinical trial designed to test if colchicine could reduce mortality and the need of invasive mechanical ventilation in patients hospitalized with COVID-19 and acute severe respiratory symptoms. The trial was organized and conducted by ECLA, which constituted an executive committee responsible for the protocol design and study oversight and for the trial coordination, and they were blinded to the trial results until database lock. PHRI helped host the database. Colchicine was donated by Spedrog Caillon to some centers lacking it. An independent data and safety monitoring board oversaw the trial and monitored the accumulating data, and they did not discuss any unblinded data with the executive committee until after database lock.

### Patients

Consenting hospitalized adults (age ≥18 years) with confirmed or suspected SARS-CoV-2 infection were eligible for the trial if they were admitted to the hospital with symptoms suggestive of COVID-19 (eg, fever or equivalent, loss of smell and/or taste, fatigue), had severe acute respiratory syndrome characterized by shortness of breath (dyspnea) or typical or atypical pneumonia on imaging, or oxygen desaturation (as measured by pulse oximetry [Spo_2_] ≤ 93%). The main exclusion criteria were clear indications or contraindications for the use of colchicine, pregnancy or breastfeeding, chronic kidney disease (creatinine clearance <15 mL/min/m^2^ [to convert to mL/s/m_2_, multiply by 0.0167]), and a negative result on a reverse transcription–polymerase chain reaction (RT-PCR) test for SARS-COV-2 available before randomization.

### Randomization and Intervention

Research electronic data capture (REDCap; Vanderbilt University) online web-based forms hosted at the Population Health Research Institute in Hamilton, Canada, were used for the concealed sequential randomization process as well as collecting hospital stay, discharge from hospital, and 28-day follow-up data.^14^ Patients were assigned in a 1:1 ratio to receive usual care or usual care plus colchicine. Randomization was restricted with blocks of random sizes and stratified by mechanical ventilation status at baseline.

Colchicine was administered orally in a loading dose of 1.5 mg immediately after randomization, followed by 0.5 mg orally within 2 hours of the initial dose, and subsequently 0.5 mg orally twice a day for 14 days or discharge, whichever occurred first. The colchicine dose was reduced in patients with kidney or liver dysfunction or if drugs that could interact were used concomitantly (eMethods in Supplement 2).

### Data Collection

We collected baseline demographic data, major clinical risk factors of known prognostic value for COVID-19, respiratory status on admission, and baseline therapies. Information regarding trial outcomes, adverse events, potential adverse reactions, and active treatment adherence was recorded during the hospital stay or up to 28 days if the patient was still hospitalized. In all patients, the vital status was ascertained up to day 28.

### Outcomes

The original trial protocol (in Supplement 1) included in-hospital mortality as the primary outcome and the composite of a new requirement for mechanical ventilation or death as a key secondary outcome. However, there was a low recruitment rate and low likelihood of reaching the original estimated sample size of 2500 patients. Contextualizing this fact in the setting of a pandemic with high mortality and morbidity rates worldwide requiring rapid and simple beneficial treatments, in November 2020, the executive committee decided to change the primary outcome prior to knowing the results to include 2 coprimary hierarchical outcomes with the aim of reducing the trial sample size and potentially getting an earlier result. Therefore, the first coprimary outcome was the composite of a new requirement for mechanical ventilation or death evaluated at 28 days after randomization. For this outcome, participants intubated at the time of randomization were only followed for death. The second coprimary outcome was death assessed at 28 days after randomization. The amended trial protocol is provided in the eMethods in Supplement 2.

Secondary outcomes were also redefined with the intention to assess more specifically the potential action of the study drug: new requirement for mechanical ventilation or death from respiratory failure, new requirement for mechanical ventilation or death from nonrespiratory cause, mortality due to respiratory failure and mortality due to nonrespiratory cause, in-hospital composite outcome, in-hospital death, mean and highest World Health Organization (WHO) descriptive score of COVID-19 during hospitalization or until 28 days (whichever came first). In patients who were not intubated at randomization, the composite outcome and death at 28 days were assessed. Deaths were centrally adjudicated at ECLA by an adjudication committee.

### Statistical Analysis

When the trial was designed, there was limited information about the rates of clinical outcomes in hospitalized patients with COVID-19. The originally estimated total sample size of 2500 patients would provide 80% power to detect a relative risk reduction of 19% in the treated group if the in-hospital mortality for the control group was 25% at a 2-sided significance level of α = .05. As the trial progressed, considering the pandemic status in Argentina and the impossibility of timely recruitment in November 2020, without knowledge of any interim result of the trial, we modified the protocol and recalculated the sample size. Assuming a 24% composite outcome (death or new intubation) at 28 days in the control group, a minimum sample size of 1200 patients would provide 80% power to detect a relative risk reduction of 27% in the treated group at a 2-sided significance level of α = .05. No α adjustments were considered since a fixed-sequence statistical approach was adopted. This strategy allows for testing each of the null hypotheses at the same significance level without any adjustment, as long as the null hypotheses to be tested are hierarchically ordered and tested in a predefined sequential order: first, the composite outcome and second, the mortality outcome.

Two formal interim analyses were planned when 28-day data on 50% and 75% of the patients were available. The monitoring boundaries specified to trigger discussion of early termination were set at 3 SDs (*P* < .0027) in the composite outcome at the 2 interim analyses (Haybittle-Peto rule). The α-level for the final analysis remained at the conventional α = .05. Efficacy analysis was conducted on an intention-to-treat basis. Cox proportional hazard regression models were used to estimate hazard ratios (HRs) and 95% CIs for coprimary and secondary outcomes evaluated at 28 days after randomization. Since the randomization was stratified by intubation status, all estimates were adjusted by this factor. For in-hospital secondary outcomes, relative risk (RR) and 95% CIs were computed. Kaplan-Meier survival curves were constructed for each group to estimate the cumulative outcome incidence as a function of time over the 28 days. Wilcoxon rank sum test was applied to compare median values between groups for COVID-19 WHO descriptive score outcomes.

Prespecified subgroup analyses were performed for the composite primary outcome according to the following subgroups defined by characteristics at randomization: age (≤60 years vs >60 years), sex, positive RT-PCR result, history of diabetes, history of hypertension, history of coronary artery disease, history of chronic lung disease, smoking status (current vs former or never smoker), use of renin-angiotensin–related medications, respiratory status, oxygen desaturation status, pneumonia at randomization, and days between admission date and randomization date. Estimated HRs with 95% CIs for each stratum are reported without adjustment for multiple comparisons. Interaction test *P* values are also reported and were computed considering Cox regression models that include an interaction term between the treatment assignment and the subgroup of interest. Although we included the *P* values, no formal conclusions can be drawn from this subgroup analysis. We estimated the primary and secondary outcomes in the per protocol population (eMethods in Supplement 2) and also conducted a post hoc analysis to estimate HRs and 95% CIs considering 2 follow-up periods: 0 to 14 days and 15 to 28 days. Statistical analyses were performed by ECLA staff using R software version 3.6.0 (R Project for Statistical Computing). Data were analyzed from June 20 to July 25, 2021. The full protocol and statistical analysis plan are listed in Supplement 1. Protocol changes are listed in the eMethods in Supplement 2.

## Results

### Patients

**Figure 1.**
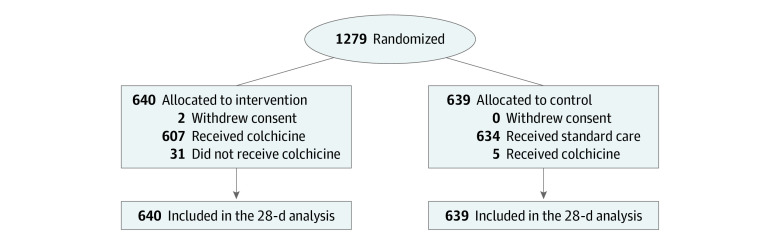
Patient Recruitment Flowchart

Between April 17, 2020, and March 28, 2021, 1279 patients (mean [SD] age, 61.8 [14.6] years; 449 [35.1%] women and 830 [64.9%] men) across 42 centers in Argentina were randomly allocated to colchicine (640 patients), the active treatment group, or usual care (639 patients), the control group (Figure 1). Two patients in the colchicine group withdrew consent. Most patients were recruited after the dexamethasone demonstrated a mortality reduction in this setting.^3^ Baseline characteristics were well balanced between the colchicine and usual care groups. More than 90% of patients reported dyspnea at entry (588 patients [92.0%] in the control group and 593 patients [92.7%] in the colchicine group), and 94% of patients were diagnosed with pneumonia (606 patients [94.8%] in the control group and 603 patients [94.2%] in the colchicine group) (Table 1). The mean (SD) oxygen saturation was 88.1% (4.8%) in the usual care group and 87.9% (5.4%) in the control group, and a total of 88 patients (6.9%) were receiving mechanical (invasive or noninvasive) ventilation when randomized. Three-quarters of the population were randomized between days 0 and 2 since hospital admission. The most common coexisting risk factor was history of hypertension, present in almost half of the patients, followed by diabetes, other cardiovascular diseases, and lung disease (Table 1). Most patients had at least 1 of the previous coexisting risk factors. A total of 1192 patients (93.2%) had a positive RT-PCR test result for SARS-CoV-2 (Table 1). The median (IQR) hospital stay was 7 (4-13) days, and the median (IQR) duration of colchicine use was 6 (3-10) days (eTable 1 in Supplement 2). All patients were followed for 28 days.

**Table 1.** Demographics and Baseline Characteristic by Group

Characteristic	Patients, No. (%) (N = 1279)	
	Usual care (n = 639)	Colchicine (n = 640)
Sex		
Women	230 (36.0)	219 (34.2)
Men	409 (64.0)	421 (65.8)
Age, mean (SD), y	62 (15)	62 (14)
Dyspnea	588 (92.0)	593 (92.7)
Pneumonia	606 (94.8)	603 (94.2)
o_2_ desaturation	518 (81.1)	515 (80.5)
o_2_ saturation, mean (SD), %	88.1 (4.8)	87.9 (5.4)
Respiratory status at randomization		
No supplemental o_2_	102 (16.0)	94 (14.7)
Noninvasive supplemental o_2_	493 (77.2)	502 (78.4)
Noninvasive mechanical ventilation	13 (2.0)	10 (1.6)
Mechanical ventilation	31 (4.9)	34 (5.3)
Time since admission, d		
0	207 (32.4)	199 (31.1)
1-2	251 (39.3)	282 (44.1)
≥3	181 (28.3)	159 (24.8)
Positive RT-PCR result	601 (94.1)	591 (92.3)
Heart rate, mean (SD), bpm	86 (17)	86 (16)
Systolic blood pressure, mean (SD), mm Hg	125 (19)	125 (19)
Respiratory rate, mean (SD), breaths/min	22.8 (9.2)	22.9 (9.0)
Body temperature, mean (SD), °C	36.81 (0.91)	36.81 (0.90)
Coexisting risk factors		
Hypertension	298 (46.6)	312 (48.8)
Diabetes	151 (23.6)	139 (21.8)
Chronic lung disease	58 (9.1)	65 (10.2)
Coronary artery disease	42 (6.6)	49 (7.7)
Heart failure	32 (5.0)	25 (3.9)
Immune suppression condition	19 (3.0)	28 (4.4)
Chronic renal disease	18 (2.8)	12 (1.9)
Active cancer	15 (2.3)	15 (2.3)
Stroke	15 (2.3)	10 (1.6)
Chronic liver disease	6 (0.9)	6 (0.9)
Smoking status		
Never	388 (61.7)	408 (64.9)
Former (last cigarette >1 y ago)	197 (31.3)	181 (28.8)
Current (last cigarette <1 y ago)	44 (7.0)	40 (6.4)
Use of renin-angiotensin related medications		
None	411 (64.3)	396 (62.0)
ACEI	122 (19.1)	119 (18.6)
AT2RB	106 (16.6)	124 (19.4)
Concomitant medication, during hospitalization		
Corticosteroids	588 (92.0)	583 (91.1)
Anticoagulant drugs	149 (23.3)	161 (25.2)
Convalescent plasma	49 (7.7)	44 (6.9)
Ivermectin	33 (5.2)	34 (5.3)
Antiplatelet drugs	23 (3.6)	30 (4.7)
Oseltamivir	8 (1.3)	10 (1.6)
Hydroxychloroquine	2 (0.3)	2 (0.3)
Lopinavir/ritonavir	1 (0.2)	1 (0.2)

Abbreviations: ACEI, angiotensin-converting enzyme inhibitor; AT2RB, angiotensin II receptor blocker; bpm, beats per minute; o_2_, oxygen; RT-PCR, reverse transcription–polymerase chain reaction.

Of 640 patients allocated to colchicine, 33 patients (5.0%) did not receive it, mainly because patients refused to take the medication after they were randomized; of 639 patients assigned to usual care, 5 patients (0.8%) received colchicine. Colchicine was not used as specified by the protocol in 92 patients (14.4%), in most cases owing to a temporary dose reduction; in 515 patients (80.5%), colchicine was received according to protocol. No patient was lost to follow-up at 28 days.

The trial allowed investigators to use the best therapeutic strategy based on the judgment of the treating physician. A total of 1171 patients (91.6%) received corticosteroids, and 310 patients (24.2%) received full-dose anticoagulation therapy. Convalescent plasma therapy was used in 93 patients (7.3%) (Table 1). Tocilizumab was not used, mainly owing to lack of it in most centers in Argentina.

### Primary Outcomes

**Figure 2.**
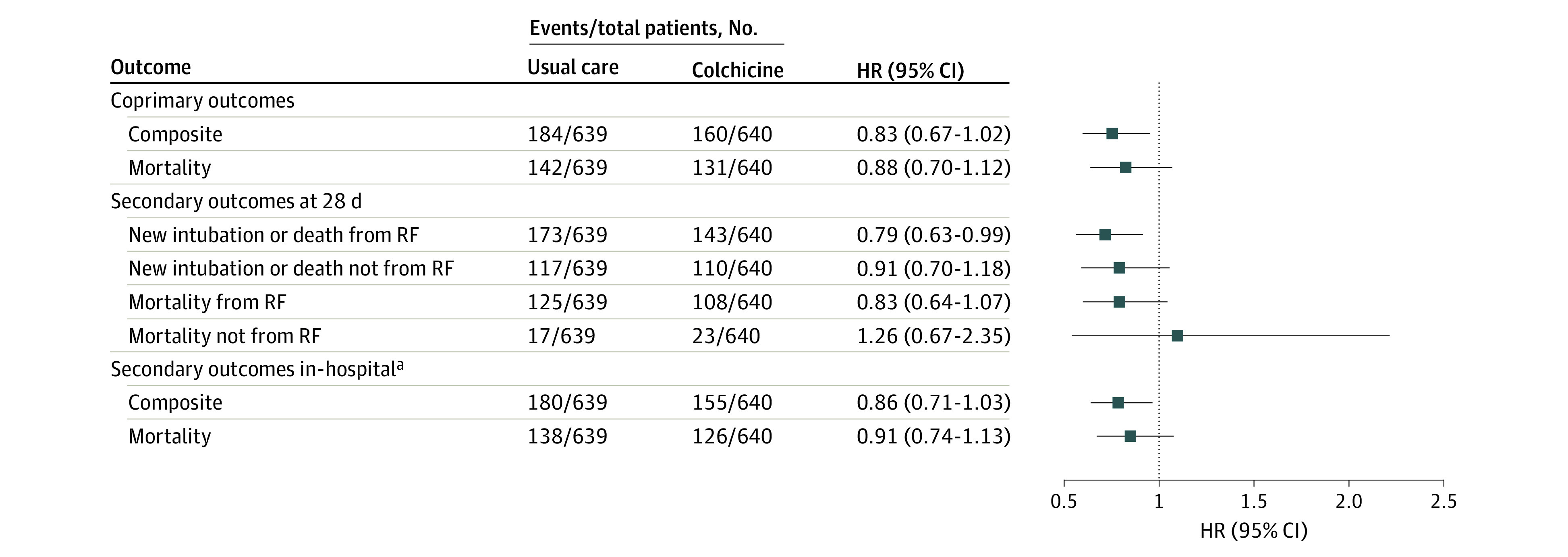
Primary and Secondary Outcomes Summary Forest Plot ^a^In-hospital outcomes are given as relative risks with 95% CIs.

The coprimary outcome of intubation for mechanical ventilation or 28-day mortality occurred in 160 patients (25.0%) in the colchicine group and 184 patients (28.8%) in the usual care group (HR, 0.83; 95% CI, 0.67-1.02; *P* = .08). Mortality at 28 days, the second coprimary outcome, occurred in 131 patients (20.5%) in the colchicine group and 142 patients (22.2%) in the usual care group (HR, 0.88; 95% CI, 0.70-1.12) (Table 2 and Figure 2).

**Table 2.** Primary and Secondary End Points

End point	No. (%)		HR (95% CI)^a^	*P* value
	Control (n = 639)	Active (n = 640)		
**Coprimary**				
28-d composite	184 (28.8)	160 (25.0)	0.83 (0.67-1.02)	.08^b^
28-d mortality	142 (22.2)	131 (20.5)	0.88 (0.70-1.12)	.30^b^
**Secondary**				
28 d				
New intubation or death				
From RF	173 (27.1)	143 (22.3)	0.79 (0.63-0.99)	.04
Not from RF	117 (18.3)	110 (17.2)	0.91 (0.7-1.18)	.49
Mortality				
From RF	125 (19.6)	108 (16.9)	0.83 (0.64-1.07)	.15
Not from RF	17 (2.7)	23 (3.6)	1.26 (0.67-2.35)	.48
In-hospital^c^				
Composite	180 (28.2)	155 (24.2)	0.86 (0.71-1.03)	.11
Mortality	138 (21.6)	126 (19.7)	0.91 (0.74-1.13)	.40
Nonintubated population, No./No. (%)^d^				
Composite	166/608 (27.3)	143/606 (23.6)	0.84 (0.67-1.05)	.13
Mortality	124/608 (20.4)	114/606 (18.8)	0.91 (0.71-1.18)	.47
COVID-19 WHO score, mean (SD)^e^				
Overall	4.00 (2.00)	4.00 (2.00)	NA	.33^f^
Highest	3.00 (2.00)	3.00 (1.00)	NA	.51^f^

Abbreviations: HR, hazard ratio; RF, respiratory failure; WHO, World Heath Organization.

^a^ No α-adjustment are considered since fixed-sequence statistical approach was adopted for multiplicity end points.

^b^ The hierarchical analysis stops after the first *P* value indicating no significance.

^c^ In-hospital secondary end points are evaluated during hospitalization (assessed up to 28 days) and relative risks and 95% CIs are presented.

^d^ Includes 1214 patients who were not intubated at randomization.

^e^ During hospitalization or until 28 days, whichever comes first.

^f^ *P* value from Wilcoxon rank-sum test.

**Figure 3.**
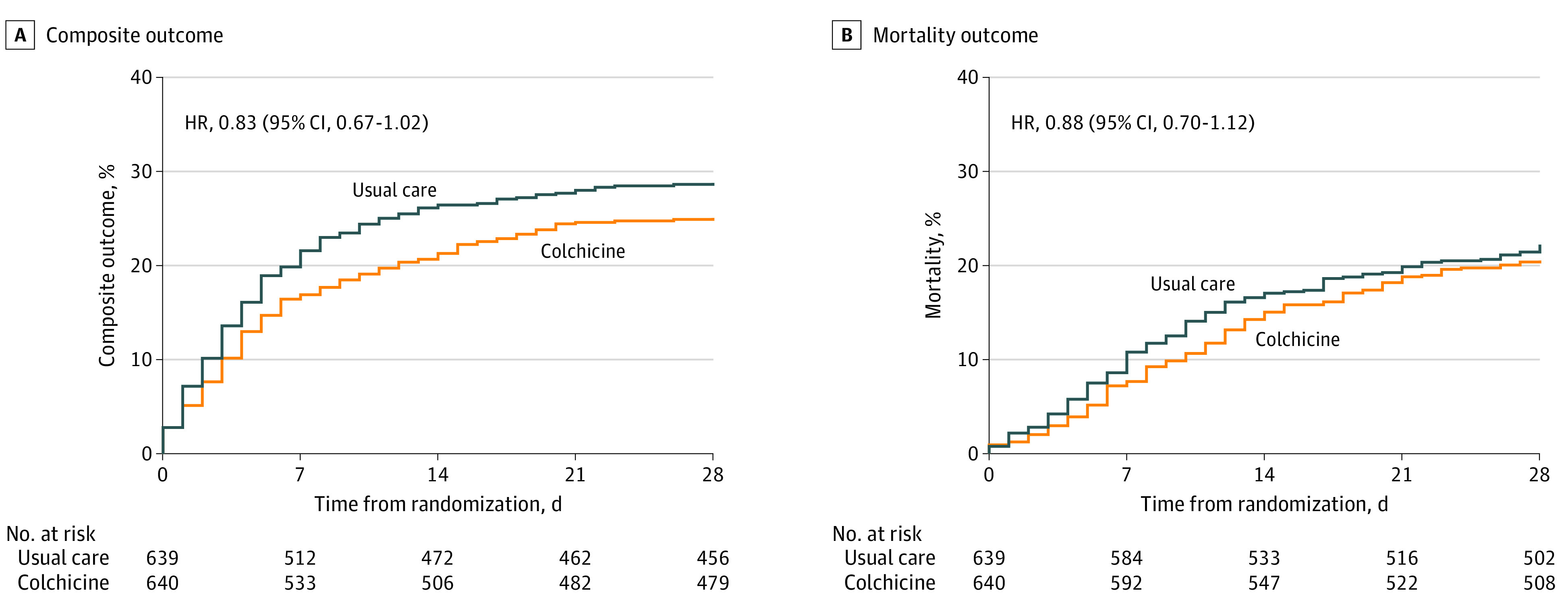
Kaplan-Meier Curves by Group for Coprimary Outcomes HR indicates hazard ratio.

As shown in Figure 3, the Kaplan-Meier curve for the combined primary outcome started to diverge as early as day 1 and continued to diverge until day 10, after which the curves started to plateau and then slightly converge when colchicine was interrupted. Most of the events occurred within the first 2 weeks after randomization. The Kaplan-Meier curve for mortality shows a slightly different pattern; although more frequent in the first weeks, the events continue to accrue until day 28. No difference in the magnitude or direction of the association was observed in any prespecified subgroup analyses (eFigure 1 in Supplement 2).

### Secondary Outcomes

The combined outcome of intubation for mechanical ventilation or 28 days mortality due to respiratory failure occurred in 143 patients (22.3%) in the colchicine group and 173 patients (27.1%) in the usual care group (HR, 0.79; 95% CI, 0.63-0.99; *P* = .04). Mortality at 28 days due to respiratory failure occurred in 108 patients (16.9%) in the colchicine group and 125 patients (19.6%) in the usual care group (HR, 0.83; 95% CI, 0.64-1.07; *P* = .15) (Table 2 and Figure 2; eTable 2 in Supplement 2).

### Per Protocol and Post Hoc Analysis

Analysis of the per protocol population found a 25% reduction in hazard of the composite outcome in favor of colchicine (HR, 0.75; 95% CI, 0.60-0.95; *P* = .02) (eFigure 2 in Supplement 2). A post hoc analysis showed that the effect of colchicine was more apparent in the first 14 days after randomization, when most of the events occurred (HR, 0.77; 95% CI, 0.61-0.96; *P* = .02), than after day 14 (HR, 1.46; 95% CI, 0.76-2.78), which supports the convergence of the Kaplan-Meier curves when treatment was stopped (eTable 3 in Supplement 2).

No differences were observed in adverse events, except severe diarrhea, which was more frequently reported in the colchicine group (69 patients [11.3%]) than the usual care group (13 patients [4.5%]) (eTable 4 and eTable 5 in Supplement 2).

## Discussion

This randomized clinical trial found that in hospitalized patients with moderate or severe COVID-19, colchicine did not improve 28-day survival or reduce the need for invasive mechanical ventilation. Importantly, our study was powered to detect a difference of 27% in that combined outcome, but instead we observed a risk difference of 17% that was not statistically significant. This might indicate a more modest benefit of colchicine in some patients, such as those included in our trial. This is supported by the lower rate of the cause-specific respiratory combined secondary outcome (new intubation or respiratory failure deaths), the rapid divergence of the first coprimary outcome Kaplan-Meier curves during the period of active treatment with a late convergence when patients in the colchicine group stopped colchicine therapy and, in a prespecified per protocol analysis, a lower rate of death or intubation at 28 days in the colchicine group.

The RECOVERY trial in patients hospitalized with COVID-19 reported no reduction in mortality with the use of colchicine.^15^ In RECOVERY, the loading dose was 50% lower than in our trial and planned treatment duration was 10 days, while in our trial, treatment duration was 14 days. Our results are consistent with the report from the COLCORONA^16^ trial, which indicated no statistically significant difference in rate of the composite of hospitalization and death in nonhospitalized patients with COVID-19. Two trials with colchicine have been published showing beneficial effects in biomarkers or clinical parameters, although they were inconclusive on impact for serious outcomes, like intubation or death.^17,18^ The ongoing ACT trial (ClinicalTrials.gov identifier: NCT04324463), which has not yet published its results, uses a high loading dose and longer treatment period.

### Limitations

This trial has some limitations. One limitations was the open-label design, but we adjudicated the events centrally without knowledge of treatment assignment. Although our trial was powered to detect a 27% risk reduction, based on the current findings, it has little statistical power (approximately 33%) to detect a more modest treatment effect.

## Conclusions

This randomized clinical trial did not demonstrate a significant benefit on 28-day mortality or intubation in hospitalized patients admitted for suspected COVID-19 pneumonia. The findings of this trial are consistent with prior larger trial in this setting.

## Notes

Supplement 1.
Trial Protocol and Statistical Analysis Plan

Supplement 2.
eMethods.
**eTable 1.** Summary Metrics of Hospital Stage and Colchicine Use Time **eTable 2.** Causes of Death by Group **eTable 3.** Post Hoc Analysis of 28-Day Composite Outcome Hazard Risk Estimation Considering Different Time Periods **eTable 4.** Summary of Adverse Events in the Safety Population **eTable 5.** Adverse Events of Special Interest Listed by System Organ Class in the Safety Population **eFigure 1.** Forest Plot for 28-Day Composite Outcome by Prespecified Subgroups **eFigure 2.** Forest Plot for Primary and Secondary Outcomes Considering the Per Protocol Population

Supplement 3.
The ECLA PHRI COLCOVID Trial Investigators

Supplement 4.
Data Sharing Statement
