# Omitted Information on Data and Safety Monitoring Board

**DOI:** 10.1001/jamanetworkopen.2022.3150

**Published:** 2022-02-18

In the Original Investigation, “Effect of Colchicine vs Usual Care Alone on Intubation and 28-Day Mortality in Patients Hospitalized With COVID-19: A Randomized Clinical Trial,”^1^ published on December 29, 2021, in *JAMA Network Open,* the names and roles of committee members and other members of the Estudios Clínicos Latino América Population Health Research Institute COLCOVID trial were inadvertently omitted from the Article Information section. In addition, the academic degrees for 3 authors were incorrect. This information and confirmation that the coauthor members of the Data and Safety Monitoring Board were not involved in the study before completion of all data collection have been corrected in the article.^1^
