# Supplementary material for: Effect of Colchicine vs Usual Care Alone on Intubation and 28-Day Mortality in Patients Hospitalized With COVID-19: A Randomized Clinical Trial
Source: JAMA Netw Open. 2021 Dec 29;4(12):e2141328. doi: 10.1001/jamanetworkopen.2021.41328 (PMC8717104; doi:10.1001/jamanetworkopen.2021.41328)
Supplement: Supplement 1. — Trial Protocol and Statistical Analysis Plan [file jamanetwopen-e2141328-s001.pdf]

Protocol

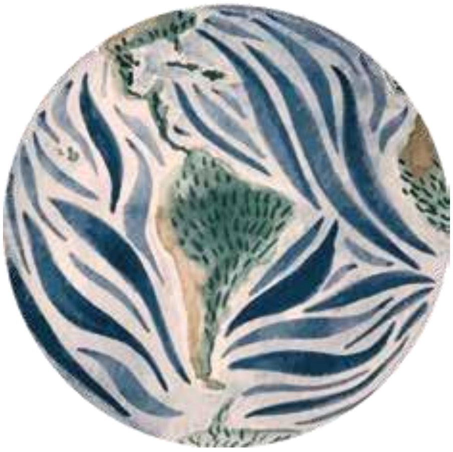

The ECLA PHRI COLCOVID Trial

A simple, pragmatic, randomized open controlled trial to test the effects of colchicine on moderate/high-risk hospitalized COVID-19 patients with the aim of reducing mortality and/or new requirement for mechanical ventilation.

Versión 2.1

Date: February, 2021

Table of contents

Background and rationale 4
Selection of patients 5
Informed Consent 5
Terminology 5
Summary of the proposal 5
Simplicity of procedures 6
Randomization 6
Colchicine dosage schedule 6
Management of patients in the study 7
Follow-up 7
Major outcomes 7
Data monitoring 7
Sample size calculation 7
Data security & Publication 8
Objectives 8
Endpoints 8
Drug discontinuation and patient withdrawalt 9
Adverse reaction reporting 9
Statistical considerations 9
Local Regulations / Declaration of Helsinki 10
Publication 10
References 10
Apendix A 12

Document History

Version	Date of release	Major modifications from previous version
1.2	19-Mar-20	Initial version (protocol V1.2)
1.2	25-Apr-20	Title – Brief Summary
2.0	30-Nov-21	Eligibility criteria<br>Co-primary outcomes<br>Secondary outcomes<br>Sample size recalculation<br>ASS - Rivaroxaban arm allowed
2.1	5-Feb-21	Secondary Outcomes specification

Background and rationale

Various anti-viral treatments are being tested in clinical trials worldwide. The WHO launched a simple, pragmatic worldwide open-label trial to test Remdesivir, Lopinavir/Ritonavir, Interferon and Hydroxy- chloroquine or Cloroquine (1).

The most important complication of COVID-19 severe cases is respiratory failure from severe acute respi- ratory syndrome (SARS), the leading cause of mortality. Accumulating evidence suggests that patients with severe COVID-19 might have a cytokine storm syndrome, a hyperinflammatory syndrome characterized by a fulminant and fatal hypercytokinemia and multiorgan failure.

The proposed pathophysiological mechanism of cytokine storm and inflammatory cascade activation is based on evidence collected primarily during the SARS-CoV and MERS-CoV epidemics (with a significant increase in IL1B, IL6, IL12, IFNγ, IP10, TNFα, IL15, and IL17 among others). The data collected during the pandemic with COVID-19 also shows a significant increase in inflammatory cytokines (GCSF, IP10, MCP1, MIP1A, and TNFα, among others) in sicker patients admitted to intensive care. In the absence of effective treatments for the management of patients with COVID-19 and respiratory failure, the immunomodulatory and anti-inflammatory effect of colchicine on cytokines involved in the hyper-inflammatory state is postulated (2). Several lines of research worldwide are testing powerful anti-inflammatory drugs for the pandemic, with different options including steroids, cytokine blockers, and other potent anti-inflam- matory agents. Steroids are partially contraindicated in viral infections.

Colchicine is a powerful anti-inflammatory drug approved for the treatment or prevention of gout and Familial Mediterranean Fever at doses ranging between 0.3 mg and 2.4 mg/day. Its mechanism of action is through the inhibition of tubulin polymerization, as well as through potential effects on cellular adhesion molecules and inflammatory chemokines. It might also have direct anti-inflammatory effects by inhibiting key inflammatory signalling networks known as inflammasome and pro-inflammatory cytokines. Additionally, evidence suggests that colchicine exerts a direct anti-inflammatory effect by inhibiting the synthesis of tumor necrosis factor alpha and IL-6, monocyte migration, and the secretion of matrix metalloproteinase-9. Through the disruption of the cytoskeleton, colchicine is believed to suppress secretion of cytokines and chemokines as well as in vitro platelet aggregation (3). All these are potentially beneficial effects that might diminish or ameliorate the COVID-19 inflammatory storm associated with severe forms of the disease. Importantly, in one contemporary trial low-dose colchicine administered to patients who survived from acute coronary syndrome shows a statistically significantly reduction of cardiovascu- lar complications (4).

We have therefore designed in a simple, pragmatic randomized controlled trial to test the effects of colchicine on severe hospitalized COVID-19 cases with the aim of reducing mortality.

Selection of patients

Inclusion Criteria (case definition)

- Consented adults (age ≥18 years) and
- COVID-19 suspicious and
- Admitted to hospital or already in hospital and
- COVID-19 suggestive symptoms (fever or febrile equivalent, loss of smell and taste, fatigue, etc.) that may be present or absent at randomization time and
- SARS (severe acute respiratory syndrome)
  - shortness of breath (dyspnea) or
  - image of typical or atypical pneumonia or
  - oxygen desaturation (SpO2 ≤ 93)

Exclusion criteria

- Clear indication or contraindication for the use of colchicine
- Pregnant or breastfeeding female.
- Chronic renal disease with creatinine clearance <15 ml/min/m2
- Negative PCR test for SARS-COV2

Informed Consent

Given the exceptional characteristics of the current pandemic, with partial (or total) restriction to access to the patient by both family members and health personnel, we have followed the International Ethical Guidelines for Health-Related Research With Human Beings (Geneva 2016) prepared by the Council of International Organizations of Medical Sciences (CIOMS) in collaboration with the World Health Organi- zation (WHO). Based on the above-mentioned guidelines, three methods of informed consent obtention will be accepted:

- Written consent
- Oral consent in the presence of two witnesses belonging to the health care team, who will document the procedure
- In case the patient cannot give consent due to a depressed conscious status, it may be included based on the decision of at least two members of the health team, one of whom must be a doctor. This will be well documented

Terminology

The novel coronavirus-induced disease first described in 2019 in China is designated COVID-19 (or COVID), and the pathogen itself (an RNA virus) is SARS-coronavirus-2 (SARS-CoV-2).

Summary of the proposal

As of March 23, 2020, 08:09 GMT, COVID-19 has been confirmed in 341,334 people worldwide and 14,746 are the associated fatalities. The mortality rate is difficult to assess due to the lack of a reliable number of cases (N), although is higher when compared to influenza outbreaks. Current focus has been on the development of novel therapeutics, including antivirals and vaccines. Nevertheless, accumulating evidence suggests that hospitalized patients with COVID-19 might have a cytokine storm

syndrome. The identification and treatment of hyperinflammation using existing and approved therapies with proven safety profiles might mitigate this associated condition and impact mortality.

The pandemic will affect our country in the near future. The wave and the exponential growth of cumulative cases have not yet arrived, and we are getting ready preparing for that contingency. We have been trained for years in a rigorous research environment. We cannot expect a more appropriate time in our professional lives to face this pandemic with what we know: investigating on the front line to improve the population health5,6.

We have worked intensively for the last days and outlined the basis of this research proposal that will attempt to mitigate the morbidity and mortality of severe cases of COVID-19. Based on the experience of China and Italy and with the help and advice of friends and experts in the area of clinical epidemiology, clinical research, cardiology, and infectious diseases, we have developed a contingency protocol to be launched in the coming days.

The rationale is to mitigate the inflammatory storm associated with severe cases of COVID-19 with the use of moderate doses of colchicine during the hospital stay of patients with severe acute respiratory syndrome (SARS) and suspicion of COVID-19, which hopefully might result in a clinical benefit.

Simplicity of procedures

To facilitate collaboration of even overcrowded hospitals, patient enrolment and randomization (via the internet) and all other trial procedures are greatly simplified, and no paperwork is required. Once a hospital has obtained approval, electronic entry of patients who have given informed consent takes only a few minutes. When entry is finished, the randomly allocated treatment is displayed on the screen and confirmed by electronic messaging.

Randomization

Those patients who meet the inclusion criteria and do not have any exclusion criteria will be centrally randomized, in a 1 to 1 ratio, to receive colchicine plus standard treatment or standard treatment. The assigned treatment will take place according to a computer-generated system. The randomization numbers of the patients will be assigned sequentially in the order in which the subjects are enrolled and will be stratified by intubation status at randomization (intubated/non intubated). Researchers will use an interactive web response system (IWRS) to obtain a treatment assignment.

Colchicine dosage schedule

The colchicine dosage schedule will vary according to the following scenarios:

- In patients not receiving Lopinavir/Ritonavir
    - Loading dose of 1.5 mg followed by 0.5 mg after two hours (day 1)
    - The next day 0.5 mg bid for 14 days or until discharge.
- In patients receiving Lopinavir/Ritonavir
    - Loading dose of 0.5 mg (day 1)

    - After 72 hours from the loading dose, 0.5 mg every 72 hours for 14 days or until discharge.
- Patients under treatment with Colchicine that are starting with Lopinavir/Ritonavir
    - Dose of 0.5 mg 72 hours after starting Lopinavir/Ritonavir.
    - Continue with 0.5 mg every 72 hours for 14 days or until discharge.

Drug interactions refer to table 1 Appendix A.

Only the oral route will be used except in the case of patients associated with mechanical ventilation or with contraindications to the oral route, in whom it will be administered by nasogastric tube.

Management of patients in the study

At all times the patient's medical team remains solely responsible for decisions about that patient's care and safety. Hence, if the team decides that deviation from the randomly allocated treatment arm is definitely necessary, this should be done.

Follow-up

Information regarding trial outcomes, adverse events, potential adverse reactions, and active treatment adherence will be recorded in-hospital and up to 28 days if the patient is still hospitalized. For patients discharged, the investigators will assess the patient's vital status on day 28.

Data to be reported include which study drugs were given (and for how many days), whether mechanical ventilation or any type of intensive care was received (and, if so, when), date of discharge, or date and cause of death. If no report is received within 6 weeks of study entry, an electronic reminder will be sent.

Drug safety Suspected unexpected serious adverse reactions that are life-threatening must be reported within 24 hours of being diagnosed, without waiting for death or discharge.

Major outcomes

The Co-primary outcomes are a Composite outcome (New requirement for mechanical ventilation or death) and mortality for all causes

Data monitoring

A global Data and Safety Monitoring Committee will keep the accumulating drug safety results and major outcome results under regular review.

Sample size calculation

A minimum sample size of 1200 patients would provide 80% power to detect a relative risk reduction of 27% in the treated group at a two-sided significance level of $\alpha = 0.05$ if a 24% 28-day composite outcome in the control group is assumed.

The ECLA PHRI COLCOVID Trial allows randomization to another trial, specifically

patients included in the trial might be (or not) randomized to an antithrombotic strategy.

Adaptive design The ECLA PHRI COLCOVID Scientific Committee may decide to add novel treatment arms while the trial is in progress. Conversely, it may decide to discontinue some treatment arms, especially if the Data and Safety Monitoring Committee reports, based on interim analyses, that one of the trial treatments definitely affects mortality.

Data security & Publication

Patient information will be encrypted. Those analysing it will use only anonymized data, and no identifiable patient details will appear in any publications. This national collaboration is coordinated through the ECLA network. Any wholly reliable interim findings will be disseminated rapidly by ECLA and published including the names of the collaborators.

Objectives

The aim of this trial is to test local standard of care plus colchicine versus local standard of care alone in moderate/severe COVID-19 hospitalized patients with the aim of reducing mortality and/or new requirement for mechanical ventilation.

Endpoints

Co-primary endpoints

The first co-primary is the composite of a new requirement for mechanical ventilation or death evaluated at 28 days after randomization. For this endpoint, participants intubated at the time of randomization will be followed for death.

The second co-primary is death assessed at 28 days after randomization.

A fixed-sequence statistical approach will be adopted. This strategy allows testing each of the null hypotheses at the same significance level alpha ($\alpha = 0.05$) without any adjustment, as long as the null hypotheses to be tested are hierarchically ordered and tested in a pre-defined sequential order: firstly the composite outcome, moving to the second co-primary endpoint (death) only after success on the first co-primary endpoint.

Secondary endpoints

1. New requirement for mechanical ventilation or death from respiratory failure
2. New requirement for mechanical ventilation or death from non-respiratory failure
3. Mortality due to respiratory failure
4. Mortality due to non-respiratory failure
5. In hospital - Composite outcome
6. In hospital - Mortality
7. Composite outcome (New requirement for mechanical ventilation or death) evaluated in Non-intubated population

8. Mortality evaluated in Non-intubated population
9. Mean WHO descriptive score of COVID-19 during hospitalization
10. Highest WHO descriptive score of COVID-19 during hospitalization

Drug discontinuation and patient withdrawalt

At all times the patient's medical team remains solely responsible for decisions about that patient's care and safety. Hence, if the medical team decides that deviation from the randomly allocated treatment arm is definitely necessary then this should be done. The colchicine administration must be stopped if the team suspects any serious unexpected drug-related reaction that is life-threatening. Patients are free to withdraw from study treatment at any time, but could still remain in the study, with in-hospital outcome reported to the study at death or discharge. Patients are also free to withdraw from the whole study at any time without any consequence and would continue to be offered the local standard of care (but would not be reported on).

Adverse reaction reporting

Any serious unexpected adverse reaction that is life-threatening must be reported through the study website within 24 hours.

Statistical considerations

Efficacy analysis will be done on an intention to-treat (ITT) basis.

Cox proportional hazard regression models wil be used to estimate Hazard Ratios (HR) and 95% confidence intervals (CI) for co-primary and secondary outcomes evaluated at 28-days post-randomization.

Since the randomization was stratified by intubation status, these estimations will be adjusted by this factor.

For in-hospital secondary outcomes, relative risk (RR) and 95% confidence intervals (CI) will computed.

Kaplan Meier survival curves will be constructed for each group to estimate the cumulative outcome incidence as a function of time over the 28 days.

Pre-specified subgroup analyses will be performed for the composite primary endpoint according to the following subgroups defined by characteristics at randomization: age (<=60 years, > 60 years), sex, positive PCR (laboratory-confirmed SARS-CoV-2throug PCR), history of diabetes, history of hypertension, history of coronary artery disease, history of chronic lung disease, smoking status (current vs. former/never), use of renin-angiotensin related medications (yes/no), respiratory status, oxygen desaturation status, pneumonia at randomization and days between admission date and randomization date.

Estimated HR with 95% CI for each stratum will be reported without adjustment for multiple comparisons. Interaction test p values will be computed considering Cox regression models that include an interaction term between the treatment assignment and the subgroup of interest. No formal conclusions will be drawn from this subgroup analysis.

For safety analyses, patients will be analyzed according to their treatment, irrespective of the random allocation. Adverse events of particular interest will be captured on the case report forms.

Sample size

When the trial was designed, there was limited information about clinical outcomes in hospitalized patients with Covid-19. The original design required a total sample size of 2500 patients, which would provide 80% power to detect a relative risk reduction of 19% in the treated group if the in-hospital mortality for the control group was 25% at a two-sided significance level of $\alpha = 0.05$.

As the trial progressed and considering the pandemic status in our country, on November 30, 2020, we decided to amend the protocol and recalculate the sample size.

Assuming a 24% 28-day composite outcome in the control group, a minimum sample size of 1200 patients would provide 80% power to detect a relative risk reduction of about 25% 30% in the treated group at a two-sided significance level of $\alpha = 0.05$. No alpha adjustments were considered since a fixed-sequence statistical approach was adopted. This strategy allows testing each of the null hypotheses at the same significance level without any adjustment, as long as the null hypotheses to be tested are hierarchically ordered and tested in a pre-defined sequential order, firstly the composite outcome, second, the mortality endpoint.

Statistical analyses will be done using R software version 3.6.0

Local Regulations / Declaration of Helsinki

The investigator will ensure that this study is carried out in full compliance with the principles of the "Declaration of Helsinki" and with the laws and regulations of the country in which the research is carried out, which provides greater protection to the individual. The study must fully adhere to the principles set forth in the "International Ethical Guidelines for Health-Related Research with Human Beings" prepared by the "Council of International Organizations of Medical Sciences (CIOMS) in collaboration with the World Health Organization (WHO).

Publication

The COLCOVID ECLA / PHRI trial will be published on behalf of the study's executive committee and all nurses and doctors who risk their lives working on the front lines in this battle to mitigate this humanitarian crisis will be listed in the appendix.

References

1. https://www.who.int/dg/speeches/detail/who-director-general-s-opening-remarks-at-the-media- briefing-on-covid-19---18-march-2020

2. Mehta P, McAuley DF, Brown M, Sanchez E, Tattersall RS, Manson JJ. COVID-19: consider cytokine storm syndromes and immunosuppression. The Lancet. doi:https://doi.org/10.1016/ S0140- 6736(20)30628-0.

3. Slobodnick A, Shah B, Krasnokutsky S, Pillinger MH. Update on colchicine, 2017. Rheumatology (Oxford). 2018; 57:i4–i11.

4. J.C. Tardif, S. Kouz, D.D. Waters, et al. Efficacy and safety of low-dose colchicine after myocardial infarction N. Engl. J. Med., 381 (2019), pp. 2497-2505

5. Yusuf S, Collins R, Peto R (1984) Why do we need some large, simple randomized trials? Stat Med 3: 409–422.

6. McDermott MM, Newman AB. Preserving Clinical Trial Integrity During the Coronavirus Pandemic. JAMA.Published online March 25, 2020. doi:10.1001/jama.2020.4689

Apendix A

Table 1 – Colchicine drug interactions

Strong P-gp inhibitor<br>Ciclosporine<br>Ranalozine	Loading dose 0.5 mg (day 1) then 0.5 mg after 72 Hs. and then 0.5 mg every 72 hs for 14 days or Until discharge
Strong/moderate CYP3A4 inhibitor<br>Lopinavir/ritonavir<br>Lopinavir<br>Ritonavir<br>Daranuvir/Ritonavir<br>Claritromicine<br>Telitromicine<br>Nelfinavir<br>Indinavir<br>Saquinavir<br>Tripanavir/Ritonavir<br>Ataznavir<br>Voxilaprevir<br>Itroconazol<br>Ketoconazol<br>Voriconazol<br>Atorvastatin<br>Simvastatin<br>Lovastatin<br>Cloranfenicol<br>Quinidine<br>Amiodarone<br>Verapamil<br>Diltiazem<br>Eritromicine<br>Fluconazol<br>Posaconazol<br>Amprenavir<br>Aprepitant<br>Fosamprenavir<br>Ciprofloxacine<br>Fenofibrate<br>Gemfibrozil<br>Grapefruit Juice<br>Pitavastatin<br>Rosuvastatin<br>Ceritinib<br>Cobicistat<br>Conivaptan<br>Dronaderone<br>Glecaprevir<br>Indinavir<br>Nefazonona	Loading dose 0.5 mg (day 1) then 0.5 mg after 72 Hs. and then 0.5 mg every 72 hs for 14 days or Until discharge

Weak CYP3A4 inhibitor<br>Azitromicine<br>Amlodipine<br>Carvedilol<br>Cilostazol<br>Naproxeno<br>Nifedipine<br>Paroxetine<br>Propafenone<br>Ticagrelor	Loading dose 1.5 mg (day 1) then 0.5 mg BID for 14 days or until discharge

Renal Failure dose adjustment

Creatinine clearance > 50 ml/min:

- Loading dose 1,5 mg follow by 0,5 mg 2 hours after the loading dose. (day 1)
- Day 2, 0,5 mg bid for 14 days or until discharge.

Creatinine clearance < 50ml/min:

- Loading dose 0,5 mg (day 1)
- New dose at 72 hours from the loading dose and then 0,5 mg every 72 hours until day 14 or until discharge.

Dosage in hepatic failure

Child-pugh A:

- Loading dose 1,5 mg follow by 0,5 mg 2 hours after the loading dose.(day 1)
- Day 2, 0,5 mg bid for 14 days or until discharge.

Child-pugh B:

- Loading dose 0,5 mg (day 1)
- New dose at 72 hours from the loading dose and then 0,5 mg every 72 hours until day 14 or until discharge.

Child-pugh C:

- Contraindicated

STATISTICAL ANALYSIS PLAN

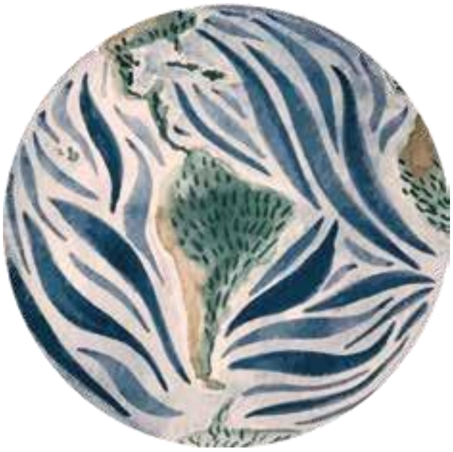

The ECLA PHRI COLCOVID Trial

Version 3

05/02/2021

Protocol Title: ECLA PHRI COLCOVID Trial
Protocol Version: Version 2.1   05/02/2021
ClinicalTrials.gov Identifier: NCT04328480
Principal Investigator: Rafael Diaz

Index

1. List of Abbreviations and Definitions of Terms 3
2. Introduction 4
3. Protocol summary 4
   3.1 Background 4
   3.2 Study objectives 4
   3.3 Treatment description 5
   3.4 Eligibility 5
   3.5 Study outcomes 6
   3.6 Sample size 6
   3.7 Randomization and Blinding 6
   3.8 Data collection 7
   3.9 Interim Analyses and Data Monitoring 7
4. General Analysis Considerations 8
   4.1 Timing of final analysis 8
   4.2 Analysis Populations 8
5. Study Data summary 8
   5.1 Subject Disposition 8
   5.2 Demographic and Baseline Variables 9
   5.3 Treatment Compliance 9
6. Analysis 10
   6.1 Outcomes definitions 10
   6.2 Analysis methods 13
   6.3 Additional analysis 14
   6.4 Pre-specified subgroup analyses 14
   6.5 Handling of Missing, Unused, and Spurious Data 14
   6.6 Confidence intervals and p-values 15
   6.7 Statistical software employed 15
7. Safety analysis 15
   7.1 Adverse events 15
   7.2 Concomitant medication 16
8. Reporting Conventions 16
9. Quality Assurance of Statistical Programming 16

Document History

Version	Date of Issue	Summary of Change
1.0	02/05/2020	Initial version – Protocol version 1.2
2.0	07/12/2020	Changes according to protocol amendment dated on November 30, 2020<br>- Sample size re recalculation<br>- Primary and secondary outcomes redefinition<br>- Pre specified subgroups analysis
3.0	05/02/2021	Changes according to protocol version 2.1 dated on February 05, 2021:<br>- Secondary Outcomes specification

1. List of Abbreviations and Definitions of Terms

Bid	Twice a day
CI	Confidence interval
DSMB	Data and Safety Monitoring Board
eCRF	Electronic case report form
FU	Follow-up
HR	Hazard risk
IQR	Inter quartile range
ITT	Intention to treat
IWRS	Interactive Web Response Systems
PCR	Polymerase chain reaction
RR	Relative risk
SARS	Severe acute respiratory syndrome
SD	Standard deviation
SpO2	Peripheral oxygen saturation
WHO	World Health Organization

2. Introduction

The purpose of this document is to describe the planned analysis and reporting for the ECLA PHRI COLCOVID Trial (NCT04328480).

This statistical analysis plan is based on the latest version of the protocol (version 2.1) dated February 05, 2021.

3. Protocol summary

3.1 Background

The most important complication of COVID-19 severe cases is respiratory failure from severe acute respiratory syndrome (SARS), the leading cause of mortality. Accumulating evidence suggests that patients with severe COVID-19 might have a cytokine storm syndrome, a hyperinflammatory syndrome characterized by a fulminant and fatal hypercytokinemia and multiorgan failure.

Colchicine, an accessible millenary drug, widely known for its anti-inflammatory properties, has potential capacities related to immunomodulation and action on cytokine release mechanisms.

The ECLA PHRI COLCOVID Trial is a simple, pragmatic randomized open controlled trial to test the effects of colchicine on moderate/high-risk hospitalized COVID-19 patients with the aim of reducing mortality and/or new requirement for mechanical ventilation.

3.2 Study objectives

Primary objectives:

- To reduce the new requirement for mechanical ventilation evaluated until 28 day or death.
- To reduce mortality evaluated until 28 day.

Secondary objectives:

- To reduce the new requirement for mechanical ventilation evaluated until 28 day or death from respiratory failure
- To reduce the new requirement for mechanical ventilation evaluated until 28 day or death from non-respiratory failure
- To reduce mortality due to respiratory failure evaluated until 28 day
- To reduce mortality due to non-respiratory failure evaluated until 28 day
- To reduce in hospital composite outcome. Evaluated during hospitalization or until death, whichever comes first, assessed up to 28 days.
- To reduce in hospital mortality. Evaluated during hospitalization or until death, whichever comes first, assessed up to 28 days
- To reduce composite outcome (New requirement for mechanical ventilation or death) evaluated in Non-intubated population.
- To reduce mortality evaluated in Non-intubated population.

- To reduce the highest WHO descriptive score of COVID-19 during hospitalization or until 28 day (whichever comes first)
- To reduce the mean WHO descriptive score of COVID-19 during hospitalization or until 28 day (whichever comes first)

3.3 Treatment description

Control Group: Local standard of care for COVID-19 SARS moderate / high-risk patients.

Active Group: Local standard of care for COVID-19 SARS moderate / high-risk patients plus colchicine.

Colchicine dosage schedule will vary according to the following scenarios:

In patients not receiving Lopinavir/Ritonavir:

- Loading dose of 1.5 mg followed by 0.5 mg after two hours (day 1)
- The next day 0.5 mg bid for 14 days or until discharge.

In patients receiving Lopinavir/Ritonavir

- Loading dose of 0.5 mg (day 1)
- After 72 hours from the loading dose, 0.5 mg every 72 hours for 14 days or until discharge.

Patients under treatment with Colchicine that are starting with Lopinavir/Ritonavir

- Dose of 0.5 mg 72 hours after starting Lopinavir/Ritonavir.
- Continue with 0.5 mg every 72 hours for 14 days or until discharge.

Only the oral route will be used except in the case of patients associated with mechanical ventilation or with contraindications to the oral route, in whom it will be administered by nasogastric tube.

3.4 Eligibility

Inclusion criteria

- Consented adults (age ≥18 years) and
- COVID-19 suspicious and
- Admitted to hospital or already in hospital and
- COVID-19 suggestive symptoms (fever or febrile equivalent, loss of smell and taste, fatigue, etc.) that may be present or absent at randomization time and
- SARS (severe acute respiratory syndrome)
    - shortness of breath (dyspnea) or
    - image of typical or atypical pneumonia or
    - oxygen desaturation (SpO2 ≤ 93)

Exclusion criteria

- Clear indication or contraindication for the use of colchicine

- Pregnant or breastfeeding
- Chronic renal disease with creatinine clearance <15 ml/min/m2
- Negative PCR test for SARS-COV2

3.5 Study outcomes

Co- Primary outcome:

- Composite outcome: New requirement for mechanical ventilation or death evaluated until 28 day.
- Mortality evaluated until 28 day.

A fixed-sequence statistical approach was adopted.

Secondary outcome:

- New requirement for mechanical ventilation or death from respiratory failure
- New requirement for mechanical ventilation or death from non-respiratory failure
- Mortality due to respiratory failure
- Mortality due to non-respiratory failure
- In hospital - Composite outcome. Evaluated during hospitalization or until death, whichever comes first, assessed up to 28 days.
- In hospital – Mortality. Evaluated during hospitalization or until death, whichever comes first, assessed up to 28 days
- Composite outcome (New requirement for mechanical ventilation or death) evaluated in Non-intubated population.
- Mortality evaluated in Non-intubated population.
- Mean WHO descriptive score of COVID-19 during hospitalization or until 28 day (whichever comes first)
- Highest WHO descriptive score of COVID-19 during hospitalization or until 28 day (whichever comes first)

3.6 Sample size

A minimum sample size of 1200 patients will provide 80% power to detect a relative risk reduction of approximately 27% in the treated group if the assumed 28 day-composite rate (new requirement of intubation and / or death) in the control group is about 24%.

3.7 Randomization and Blinding

Randomization will be via central IWRS. Randomization will occur in 1:1 ratio to colchicine vs. control, stratified by intubation status (intubated/ non intubated) at randomization and using randomly permuted blocks (blocks sizes: 4, 6 and 8).

This is an open-label study. However, while the study is in progress, access to tabular results of study outcomes by treatment allocation will not be available to the Executive

Committee. The Data and Safety Monitoring Board (DSMB) and the DSMB reporting statisticians will be unblinded.

3.8 Data collection

Baseline and follow-up information will be collected on trial-specific electronic case report forms (eCRFs) through REDCap web application.

All randomized participants will be followed up until death or 28 days post-randomization.

3.9 Interim Analyses and Data Monitoring

The DSMB will review, safety events, including outcome events, serious adverse events, and other adverse events. The DSMB Charter is describe in the document "DSMB Charter _COLCOVID trial_v2.0.docx".

For efficacy, two formal interim analyses were planned when 50% and 75% of the patients have completed followed up. A suggested guideline for early termination is a reduction of 3 standard deviations (p-value<0.0027) in the composite outcome at the two interim analysis (Haybittle-Peto rule). The α-level for the final analysis will remain at the conventional $\alpha = 0.05$.

The DSMB will monitor for an adverse impact of colchicine. For safety, two formal reviews were planned when 25% and 50% of the patients have completed followed up. A suggested guideline of a 3 standard deviations excess in the first analysis and a 2 standard deviations excess in the second analysis would trigger discussions about stopping for harm. This safety stopping guideline does not preclude the DSMB from making recommendations at any time in the context of patient safety.

4. General Analysis Considerations

4.1 Timing of final analysis

Final analysis is planned to take place in two stages:

The first efficacy report will be prepared when the sample size of 1200 patients is reached and every patient has reached at least 10/15 days follow-up and data for the primary endpoint has been received and cleaned.

The main report/publication of the trial will be prepared when the total sample size of 1200 patients is reached and every patient has reached 28 days follow-up and data for the primary endpoint has been received and cleaned.

4.2 Analysis Populations

- *Intention to treat Population (ITT):* All randomized patients regardless of their eligibility, according to the treatment they were randomized to receive. Patients with no data available after randomization are excluded from this analysis set. This population will be used for efficacy data analysis.
- *Safety population:* All randomized patients who have received study treatments (active or control). Patients will be analyzed according to the treatment they actually received. Patients with no data available after randomization are excluded from this analysis set. This population will be used for safety data analysis.
- *Per protocol population*: All randomized patients who have received study treatments (active or control). Patients will be analyzed within the intervention group to which they were randomized after exclusion of non-compliant patients. Non-compliant patients for colchicine group are defined as patients who do not received colchicine or patients who have received colchicine but discontinued. Non-compliant patients for standard care group are defined as patients who received colchicine.

5. Study Data summary

All continuous variables will be summarized using the following descriptive statistics: mean, standard deviation (SD) and range if data presented a normal distribution and median, inter quartile range (IQR) and range if data skewed. The frequency and percentages (based on the non-missing sample size) of observed levels will be reported for all categorical measures. All summary tables will be structured with a column for each treatment in the order (Control, Active) and will be annotated with the total population size relevant to that table/treatment, including any missing observations.

5.1 Subject Disposition

A CONSORT flow diagram will be used to summarize the number of patients who were:

- randomized
- allocated to each arm

- withdrew consent at each arm
- received the randomized allocation at each arm
- did not received the randomized allocation at each arm
- completed study through day 28 at each arm
- did not completed study through day 28 at each arm
- lost to follow-up at each arm
- included in primary analysis

5.2 Demographic and Baseline Variables

The following characteristics will be summarized:

- Sex
- Age at randomization
- Dyspnea status at randomization time
- Pneumonia at randomization time
- Oxygen desaturation at randomization time
- Oxygen desaturation level at randomization time
- Respiratory status at randomization time
- Physical status at randomization time (Heart rate, Systolic blood pressure, Respiratory rate, Body temperature)
- Comorbidities (Diabetes, Hypertension, Coronary artery disease, Heart failure, Stroke, Immune suppression condition, Chronic Lung Disease, Smoking status, Chronic renal disease, Chronic liver disease, Active cancer)

5.3 Treatment Compliance

The number and proportion of patients who did not receive the treatment they were allocated to will be reported.

Treatment compliance for Colchicine arm will be assessed considering the percentage of days that the patient received colchicine. The protocol specifies the treatment duration at 14 days or until discharge.

6. Analysis

6.1 Outcomes definitions

7.1.1 Co-Primary outcome #1

Composite outcome: New requirement for mechanical ventilation evaluated until 28 day or death. (Worsening patients status)

<u>Outcome definition:</u>

- For patients who were not intubated at randomization (adm_respstatus<4) this outcome is defined as the new requirement for mechanical ventilation at any moment during the period between 2 hours after randomization and 28 days FU or the occurrence of death from any cause during 28 days FU.
- For patients who were intubated at randomization or within 2 hours after randomization (adm_respstatus=4) this outcome is defined as the occurrence of death from any cause during 28 days FU.

New intubations that have occurred after 28 days FU and Deaths that have occurred after 28 days FU are not considered.

Possible values: occurred/non occurred

<u>Time –to- event computations:</u>

- For patients who were not intubated at randomization (adm_respstatus<4) time to composite outcome is defined as the time since randomization date to the new requirement for mechanical ventilation date or the death date (the first one during 28 days FU).
- For patients who were intubated at randomization or within 2 hours after randomization (adm_respstatus=4) time to composite outcome is defined as the time since randomization to the death date (during 28 days FU).

Patients who did not experience the outcome will be censored at day 28 or at the last day with available information (before day 28).

7.1.2 Co-Primary outcome #2

Mortality (all causes)

<u>Outcome definition:</u>

For all patients this outcome is defined as the occurrence of death from any cause during 28 days FU. Deaths that have occurred after this period are not considered.

Possible values: occurred/non occurred

<u>Time –to- event computations:</u>

For all patients, time to death outcome is defined as the time since randomization to the death date (during 28 days FU). Patients who did not experience the outcome will be censored at day 28 or at the last day with available information (before day 28).

7.1.3 Secondary outcome #1

New requirement for mechanical ventilation or death from respiratory failure

<u>Outcome definition:</u>

- For patients who were not intubated at randomization (adm_respstatus<4) this outcome is defined as the new requirement for mechanical ventilation at any moment during the period between 2 hours after randomization and 28 days FU or the occurrence of death from respiratory failure during 28 days FU.
- For patients who were intubated at randomization or within 2 hours after randomization (adm_respstatus=4) this outcome is defined as the occurrence of death from respiratory failure during 28 days FU.

New intubations that have occurred after 28 days FU and Deaths that have occurred after 28 days FU are not considered.

Possible values: occurred/non occurred

7.1.4 Secondary outcome #2

New requirement for mechanical ventilation or death from non-respiratory failure

<u>Outcome definition:</u>

- For patients who were not intubated at randomization (adm_respstatus<4) this outcome is defined as the new requirement for mechanical ventilation at any moment during the period between 2 hours after randomization and 28 days FU or the occurrence of death from non-respiratory failure during 28 days FU.
- For patients who were intubated at randomization or within 2 hours after randomization (adm_respstatus=4) this outcome is defined as the occurrence of death from non- respiratory failure during 28 days FU.

New intubations that have occurred after 28 days FU and Deaths that have occurred after 28 days FU are not considered.

Possible values: occurred/non occurred

7.1.5 Secondary outcome #3

Mortality due to respiratory failure

<u>Outcome definition:</u>

For all patients this outcome is defined as the occurrence of death from respiratory failure during 28 days FU. Deaths that have occurred after this period are not considered.

Possible values: occurred/non occurred

7.1.6 Secondary outcome #4

Mortality due to non-respiratory failure

Outcome definition:

For all patients this outcome is defined as the occurrence of death from non-respiratory failure during 28 days FU. Deaths that have occurred after this period are not considered.

Possible values: occurred/non occurred

7.1.7 Secondary outcome #5

In-hospital - Composite outcome:

Outcome definition:

- For patients who were not intubated at randomization (adm_respstatus<4) this outcome is defined as the new requirement for mechanical ventilation at any moment during the period between 2 hours after randomization and discharge (assessed up to 28 days) or the occurrence of death from any cause during hospitalization (assessed up to 28 days).
- For patients who were intubated at randomization or within 2 hours after randomization (adm_respstatus=4) this outcome is defined as the occurrence of death from any cause during hospitalization (assessed up to 28 days).

New intubations that have occurred after 28 days FU and Deaths that have occurred after 28 days FU and/or out-hospital are not considered.

Possible values: occurred/non occurred

7.1.8 Secondary outcome #6

In-hospital - Mortality

Outcome definition:

For all patients this outcome is defined as the occurrence of death from any cause during hospitalization (assessed up to 28 days). Deaths that have occurred after 28 days FU and/or out-hospital are not considered.

Possible values: occurred/non occurred

7.1.9 Secondary outcome #7

Composite outcome evaluated in Non-intubated population.

Outcome definition:

This outcome is define as was described in point "7.1.1 Co-Primary outcome #1" and will be evaluated in the Non-intubated population (adm_respstatus<4).

7.1.10 Secondary outcome #8

Mortality evaluated in Non-intubated population.

Outcome definition:

This outcome is define as was described in point "7.1.2 Co-Primary outcome #2" and will be evaluated in the Non-intubated population (adm_respstatus<4).

7.1.11 Secondary outcome #19

Mean WHO descriptive score of COVID-19

<u>Outcome definition:</u>

For each patient this outcome is defined as the mean value of the WHO descriptive COVID-19 score observed during the hospitalization or until 28 day (whichever comes first). Baseline score value is considered in the computation.

For patients who discharge alive from hospital, score=2 (ambulatory - limitation of activities) will be adjudicated.

Possible values: 2.5 - 7

Table 1. Ordinal Scale for Clinical Improvement (WHO)

Patient state	Descriptor	Score
**Uninfected**	No clinical or virological evidence of infection	0
**Ambulatory**	No limitation of activities	1
	Limitation of activities	2
**Hospitalized mild disease**	Hospitalized, no oxygen therapy	3
	Oxygen by mask or nasal prongs	4
**Hospitalized severe disease**	Non-invasive ventilation or high-flow oxygen	5
	Intubation and mechanical ventilation	6
	Ventilation + additional organ support - pressors, RRT, ECMO	7
**Death**	Death	8

7.1.12 Secondary outcome #10

Highest WHO descriptive score of COVID-19

<u>Outcome definition:</u>

For each patient this outcome is defined as the highest value of the WHO descriptive COVID-19 score observed during the hospitalization or until 28 day (whichever comes first).

Baseline score value is considered in the computation.

For patients who discharge alive from hospital, score=2 (ambulatory - limitation of activities) will be adjudicated.

Possible values: 3 - 8

6.2 Analysis methods

For all outcomes, ITT population will be considered and therefore comparisons will be made under the intention-to-treat principle.

Co-primary outcomes (composite and mortality) will be summarized with counts and percentages by randomized group.

Kaplan-Meier estimates curves for the time to event will be plotted.

Hazard risk (HR) and 95% Confidence Interval (CI) will be estimated through a Cox proportional hazard model. Adjustment by intubation status at randomization time will be considered.

In hospital- composite outcome and in-hospital mortality will be summarized with counts and percentages by randomized group. Relative risk (RR) and 95% CI will be estimated.

COVID-19 WHO descriptive score outcomes will be summarized with mean, SD, median and IQR by randomized group. T-student test and Wilcoxon test will be applied to compare the mean (or median) between groups.

6.3 Additional analysis

Adjustment for relevant baseline characteristics will be incorporated if important imbalances between the randomized groups are observed in baseline subgroups.
This will be done, for the co-primary outcome#1, through the incorporation of the corresponding term(s) in the Cox regression model in order to obtain adjusted hazard ratio estimation.

6.4 Pre-specified subgroup analyses

Pre-specified subgroup analyses will be conducted for the composite outcome.
Results will be presented on forest plots as HR with 95% CI. The following subgroups will be examined:

- Age (<mean age, > mean age)
- Time since hospital admission date to randomization date
- Sex (female, male)
- Positive PCR at randomization (yes/no)
- Pneumonia at baseline (yes/no)
- Hemoglobin saturation at baseline (<93% vs. >93%).
- Mechanical ventilation at randomization (yes/no)
- Diabetes (yes/no)
- Hypertension (yes/no)
- Coronary artery disease (yes/no)
- Chronic Lung Disease (yes/no)
- Smoking status (current vs former/never)
- Use of renin-angiotensin related medications (yes/no)

6.5 Handling of Missing, Unused, and Spurious Data

All reasonable attempts will be made to ensure the integrity of the data collection of this study.
Variables with more than 20% of missing data will not be included in the analysis. For variables that have less than 20% of missing values, firstly it will be investigated and tried to complete the missing data. Available data will be included in the data listings

and tabulations showing for each report the actual total. No imputation techniques will be used for missing data.

6.6 Confidence intervals and p-values

All applicable statistical tests will be 2-sided and will be performed using a 5% significance level.
All confidence intervals presented will be 95% and two-sided

Multiplicity adjustment

A fixed-sequence statistical approach was adopted. This strategy allows testing of each of the null hypotheses at the same significance level without any adjustment, as long as the null hypotheses to be tested are hierarchically ordered and tested in a pre-defined sequential order.
In this approach all tests are evaluated at the same significance level alpha ($\alpha = 0.05$), moving to a second endpoint only after a success on the previous endpoint. This test procedure does not inflate the type I error rate as long as there is (1) prospective specification of the testing sequence and (2) no further testing once the sequence breaks, that is, further testing stops as soon as there is a failure of an endpoint in the sequence to show significance at level alpha ($\alpha = 0.05$).
Pre-defined sequential order:
(1) Composite outcome: New requirement for mechanical ventilation or death evaluated until 28 day.
(2) Mortality evaluated until 28 day.

6.7 Statistical software employed

The statistical software R version 3.6.0 will be used for the interim and final analyses.

7. Safety analysis

7.1 Adverse events

Adverse Events of Special Interest will be captured on the case report forms and will be exempted from expedited reporting.
Suspected unexpected life-threatening serious adverse reactions should be reported within 24 hours, without waiting for death or discharge.
Serious adverse events with a reasonable causal relationship to study interventions, unexpected for the patient population under study or inconsistent with the product information (i.e., SUSARs), and that occur in participants treated with study medication will be reported to the regulatory agencies on CIOMS forms) within the required timelines.
All adverse events that lead to permanent discontinuation of study interventions will be recorded and included in the final study report.

A table of all adverse events of special interest will be presented with counts and percentages by group. Safety population will be considered for this analysis and relative risk and 95% CI will be estimated.

A table of all adverse events that lead to permanent discontinuation of study intervention will be presented with counts and percentages for Colchicine arm. Safety population will be considered for this analysis.

7.2 Concomitant medication

A table of concomitant medications will be presented with counts and percentages by group. Safety population will be considered for this analysis.

8. Reporting Conventions

P-values ≥0.001 will be reported to 3 decimal places; p-values less than 0.001 will be reported as "<0.001". The mean, standard deviation, and any other statistics other than quantiles, will be reported to one decimal place greater than the original data. Quantiles, such as median, or minimum and maximum will use the same number of decimal places as the original data. Estimated parameters, not on the same scale as raw observations will be reported to 3 significant figures.

9. Quality Assurance of Statistical Programming

A second review statistician will independently reproduce the interim and final analyses and summary statistics tables.
