# Supplementary material for: Effect of Colchicine vs Usual Care Alone on Intubation and 28-Day Mortality in Patients Hospitalized With COVID-19: A Randomized Clinical Trial
Source: JAMA Netw Open. 2021 Dec 29;4(12):e2141328. doi: 10.1001/jamanetworkopen.2021.41328 (PMC8717104; doi:10.1001/jamanetworkopen.2021.41328)
Supplement: Supplement 2. — eMethods. eTable 1. Summary Metrics of Hospital Stage and Colchicine Use Time eTable 2. Causes of Death by Group eTable 3. Post Hoc Analysis of 28-Day Composite Outcome Hazard Risk Estimation Considering Different Time Periods eTable 4. Summary of Adverse Events in the Safety Population eTable 5. Adverse Events of Special Interest Listed by System Organ Class in the Safety Population eFigure 1. Forest Plot for 28-Day Composite Outcome by Prespecified Subgroups eFigure 2. Forest Plot for Primary and Secondary Outcomes Considering the Per Protocol Population [file jamanetwopen-e2141328-s002.pdf]

Supplemental Online Content

Diaz R, Orlandini A, Castellana N, et al; the ECLA PHRI COLCOVID Trial Investigators. Effect of colchicine vs usual care alone on intubation and 28-day mortality in patients hospitalized with COVID-19: a randomized clinical trial. *JAMA Netw Open*. 2021;4(12):e2141328. doi:10.1001/jamanetworkopen.2021.41328

eMethods.
eTable 1. Summary Metrics of Hospital Stage and Colchicine Use Time
eTable 2. Causes of Death by Group
eTable 3. Post Hoc Analysis of 28-Day Composite Outcome Hazard Risk Estimation Considering Different Time Periods
eTable 4. Summary of Adverse Events in the Safety Population
eTable 5. Adverse Events of Special Interest Listed by System Organ Class in the Safety Population
eFigure 1. Forest Plot for 28-Day Composite Outcome by Prespecified Subgroups
eFigure 2. Forest Plot for Primary and Secondary Outcomes Considering the Per Protocol Population

This supplemental material has been provided by the authors to give readers additional information about their work.

© 2021 Diaz R et al. *JAMA Network Open.*

eMethods.

Study organization

The trial was conducted at 42 Argentinian hospitals. The trial was coordinated by ECLA (Estudios Clinicos Latino America), the trial sponsor and with the collaboration of PHRI (Population Health Research Institute).
Treatment supply to local sites was supported by Spedrong Caillion Pharma and registry data was supported by Red Cap.

Protocol changes

COLCOVID is a randomized trial among patients hospitalized for COVID-19. All eligible patients receive usual standard of care in the participating hospital and are randomly allocated between no additional treatment and one active treatment arm.
The original and final protocols are included in the supplementary material to this publication, together with summaries of the changes made.
The following modifications were made to the protocol during the trial:

Version	Date of release	Major modifications from previous version
1.2	19-Mar-20	Initial version (protocol V1.2)
1.2	25-Apr-20	Title – Brief Summary
2.0	30-Nov-21	Eligibility criteria<br>Co-primary outcomes<br>Secondary outcomes<br>Sample size recalculation<br>ASS - Rivaroxaban arm allowed
2.1	5-Feb-21	Secondary Outcomes specification

© 2021 Diaz R et al. *JAMA Network Open.*

Patients informed consents

Three methods of obtaining informed consent were accepted by our local health authorities (ANMAT):
a) Written consent
b) Oral consent in the presence of two witnesses belonging to the health team, who formally documented this act
c) If the patient could not give consent due to cognitive disfunction, and a legal representative was not available, the patient could be included based on the decision of at least two members of the health team, one of whom must be a doctor.

Confirmed positive PCR

Confirmed positive PCR is defined as Laboratory-confirmed SARS-CoV-2throug PCR at randomization or pending result at randomization and positive result confirmed later

Per protocol population

All randomized patients who have received study treatments (active or control) are included in this population. Patients are analyzed within the intervention group to which they were randomized after exclusion of non-compliant patients. Non-compliant patients for colchicine group were defined as patients who did not received colchicine or patients who received colchicine but discontinued. Non-compliant patients for standard care group were defined as patients who received colchicine.

Colchicine administration

The colchicine dosage schedule will vary according to the following scenarios:

1. In patients not receiving Lopinavir/Ritonavir
   - Loading dose of 1.5 mg followed by 0.5 mg after two hours (day 1)
   - The next day 0.5 mg bid for 14 days or until discharge.
2. In patients receiving Lopinavir/Ritonavir
   - Loading dose of 0.5 mg (day 1)
   - After 72 hours from the loading dose, 0.5 mg every 72 hours for 14 days or until discharge.
3. Patients under treatment with Colchicine that are starting with Lopinavir/Ritonavir
   - Dose of 0.5 mg 72 hours after starting Lopinavir/Ritonavir.
   - Continue with 0.5 mg every 72 hours for 14 days or until discharge.

Only the oral route will be used except in the case of patients associated with mechanical ventilation or with contraindications to the oral route, in whom it will be administered by nasogastric tube.

Colchicine drug interactions

© 2021 Diaz R et al. *JAMA Network Open.*

Strong P-gp inhibitor	Loading dose 0.5 mg (day 1) then 0.5 mg after 72
Ciclosporine	Hs. and then 0.5 mg every 72 hs for 14 days or
Ranalozine	Until discharge
Strong/moderate CYP3A4 inhibitor	Loading dose 0.5 mg (day 1) then 0.5 mg after 72
Lopinavir/ritonavir	Hs. and then 0.5 mg every 72 hs for 14 days or
Lopinavir	Until discharge
Ritonavir	
Daranuvir/Ritonavir	
Claritromicine	
Telitromicine	
Nelfinavir	
Indinavir	
Saquinavir	
Tripanavir/Ritonavir	
Ataznavir	
Voxilaprevir	
Itroconazol	
Ketoconazol	
Voriconazol	
Atorvastatin	
Simvastatin	
Lovastatin	
Cloranfenicol	
Quinidine	
Amiodarone	
Verapamil	
Diltiazem	
Eritromicine	
Fluconazol	
Posaconazol	
Amprenavir	
Aprepitant	
Fosamprenavir	
Ciprofloxacine	
Fenofibrate	
Gemfibrozil	
Grapefruit Juice	
Pitavastatin	
Rosuvastatin	
Ceritinib	
Cobicistat	
Conivaptan	
Dronaderone	
Glecaprevir	
Indinavir	

© 2021 Diaz R et al. *JAMA Network Open.*

Nefazonona	
Weak CYP3A4 inhibitor	Loading dose 1.5 mg (day 1) then 0.5 mg BID for
Azitromicine	14 days or until discharge
Amlodipine	
Carvedilol	
Cilostazol	
Naproxeno	
Nifedipine	
Paroxetine	
Propafenone	
Ticagrelor	

Renal failure dose adjustment

Creatinine clearance > 50 ml/min:

- Loading dose 1.5 mg follow by 0.5 mg 2 hours after the loading dose. (day 1)
- Day 2, 0.5 mg bid for 14 days or until discharge.

Creatinine clearance < 50ml/min:

- Loading dose 0.5 mg (day 1)
- New dose at 72 hours from the loading dose and then 0.5 mg every 72 hours until day 14 or until discharge.

Dosage in hepatic failure

Child-pugh A:

- Loading dose 1.5 mg follow by 0.5 mg 2 hours after the loading dose. (day 1)
- Day 2, 0.5 mg bid for 14 days or until discharge.

Child-pugh B:

- Loading dose 0.5 mg (day 1)
- New dose at 72 hours from the loading dose and then 0.5 mg every 72 hours until day 14 or until discharge.

Child-pugh C:

- Contraindicated

© 2021 Diaz R et al. *JAMA Network Open.*

eTable 1. Summary Metrics of Hospital Stage and Colchicine Use Time

	Median	P25%	P75%
Hospital stage time (days)	7	4	13
Colchicine use time (days)	6	3	10

eTable 2. Causes of Death by Group

Death cause	Randomization Group	
	Usual Care<br>N = 639	Colchicine<br>N = 640
Respiratory failure, n (%)	125 (19.6)	108 (16.9)
Cardiovascular, n (%)	7 (1.1)	6 (1.2)
Major bleeding, n (%)	0 (0.0)	2 (0.3)
Septic shock, n (%)	8 (1.3)	10 (1.6)
Trauma, n (%)	0 (0.0)	4 (0.6)
Unknown origin, n (%)	2 (0.3)	1 (0.2)

eTable 3. Post Hoc Analysis of 28-Day Composite Outcome Hazard Risk Estimation Considering Different Time Periods

Time period	HR	95% CI	p-value
0 to 14 days	0.77	[0.61–0.96]	0.023
15 to 28 days	1.46	[0.76–2.78]	0.250

© 2021 Diaz R et al. *JAMA Network Open.*

eTable 4. Summary of Adverse Events in the Safety Population

**Adverse Event**	**Usual care N = 665**	**Colchicine N = 612**
At least one adverse event, n /N (%)	229 /665 (34.5)	245 /612 (40.0)
Severe lymphopenia, n /N (%)	98 /661 (14.8)	92 /610 (15.1)
Severe anemia, n /N (%)	31 /661 (4.7)	25 /610 (4.1)
Severe thrombocytopenia, n /N (%)	16 /660 (2.4)	21 /610 (3.4)
Major bleeding, n /N (%)	6 /661 (0.9)	6 /610 (1.0)
Disseminated intravascular coagulation, n /N (%)	3 /661 (0.5)	3 /610 (0.5)
Cardiac arrest, n /N (%)	43 /661 (6.5)	41 /610 (6.7)
Heart failure, n /N (%)	32 /661 (4.8)	20 /610 (3.3)
Myocarditis, n /N (%)	4 /661 (0.6)	4 /610 (0.7)
Myocardial infarction, n /N (%)	5 /661 (0.8)	1 /610 (0.2)
ECMO, n /N (%)	4 /661 (0.6)	0 /610 (0.0)
Sepsis, bacterial suspected, n /N (%)	104 /661 (15.7)	102 /610 (16.7)
Septic shock, n /N (%)	74 /661 (11.2)	67 /610 (11.0)
Acute kidney injury, n /N (%)	53 /661 (8.0)	51 /610 (8.4)
Renal replacement therapy, n /N (%)	11 /661 (1.7)	13 /610 (2.1)

© 2021 Diaz R et al. *JAMA Network Open.*

Adverse Event	Usual care N = 665	Colchicine N = 612
Severe diarrhea, n /N (%)[a]	13 /288 (4.5)	69 /608 (11.3)
GI bleeding, n /N (%)	0 /665 (0.0)	2 /612 (0.3)
Laboratory abnormality, n /N (%)	0 /665 (0.0)	4 /612 (0.7)
Pulmonary embolism, n /N (%)	5 /661 (0.8)	6 /610 (1.0)
Stroke, n /N (%)[a]	2 /324 (0.6)	1 /303 (0.3)
Deep venous thrombosis, n /N (%)[a]	1 /323 (0.3)	1 /302 (0.3)
Acute limb ischemia, n /N (%)[a]	0 /324 (0.0)	1 /304 (0.3)

Adverse events that occurred in more than 1 patient after randomization through day 28 are shown.
Percentages are based on the safety population, which comprised all the patients who underwent randomization and who received study treatments. Patients are analyzed according to the treatment they actually received
[a] Stroke, Deep venous thrombosis and acute limb ischemia questions were incorporated in eCRF on January 01 2021. Severe diarrhea for Usual Caregroup question was also incorporated at the same time

© 2021 Diaz R et al. *JAMA Network Open.*

eTable 5. Adverse Events of Special Interest Listed by System Organ Class in the Safety Population

System Organ Class	Usual care N = 665	Colchicine N = 612
At least one adverse event, n (%)	229 (34.4)	245 (40.0)
Blood and lymphatic system disorders, n (%)	114 (17.1)	112 (18.3)
Infections and infestations, n (%)	116 (17.5)	110 (18.0)
Cardiac disorders, n (%)	74 (11.2)	54 (8.8)
Renal and urinary disorders, n (%)	56 (8.4)	51 (8.3)
Gastrointestinal disorders, n (%)	13 (1.9)	70 (11.4)
Vascular disorders, n (%)	8 (1.2)	9 (1.5)
Investigations, n (%)	0 (0.0)	4 (0.7)

© 2021 Diaz R et al. *JAMA Network Open.*

eFigure 1. Forest Plot for 28-Day Composite Outcome by Prespecified Subgroups

Subgroup	Usual Care	Colchicine	HR [95% CI]	P-value[a]
**Overall**	184 / 639	160 / 640	0.83 [0.67, 1.02]	0.08
**Age**				0.52
<= 60	47 / 270	40 / 279	0.73 [0.48, 1.12]	
> 60	137 / 369	120 / 361	0.87 [0.68, 1.11]	
**Sex**				0.09
Male	65 / 230	66 / 219	0.71 [0.54, 0.93]	
Female	119 / 409	94 / 421	1.05 [0.74, 1.48]	
**Positive PCR**				0.83
No	12 / 38	14 / 49	0.86 [0.39, 1.88]	
Yes	172 / 601	146 / 591	0.82 [0.66, 1.02]	
**History of diabetes**				0.86
No	131 / 488	119 / 500	0.84 [0.66, 1.08]	
Yes	53 / 151	41 / 139	0.79 [0.53, 1.20]	
**History of hypertension**				0.3
No	85 / 341	65 / 327	0.73 [0.52, 1.01]	
Yes	99 / 298	95 / 312	0.91 [0.68, 1.20]	
**Coronary artery disease**				0.79
No	171 / 597	146 / 590	0.82 [0.66, 1.02]	
Yes	13 / 42	14 / 49	0.97 [0.45, 2.12]	
**Chronic lung disease**				1
No	165 / 581	144 / 574	0.84 [0.67, 1.05]	
Yes	19 / 58	16 / 65	0.74 [0.38, 1.44]	
**Smoking status**				0.86
Never/Former	166 / 585	149 / 589	0.85 [0.68, 1.06]	
Current	15 / 44	11 / 40	0.75 [0.34, 1.63]	
**Use of renin-angiotensin related medications**				0.41
No	111 / 411	89 / 396	0.75 [0.57, 1.00]	
Yes	73 / 228	71 / 243	0.89 [0.64, 1.24]	
**Respiratory Status**				0.6
No supplemental O2	13 / 102	6 / 93	0.48 [0.18, 1.27]	
Non-invasive supplemental O2	145 / 493	131 / 502	0.86 [0.68, 1.09]	
Non-invasive mechanical ventilation	8 / 13	6 / 10	1.21 [0.42, 3.49]	
Mechanical Ventilation	18 / 31	17 / 34	0.70 [0.36, 1.37]	
**Oxygen desaturation**				0.78
No	14 / 121	11 / 125	0.74 [0.34, 1.64]	
Yes	170 / 518	149 / 515	0.84 [0.67, 1.04]	
**Pneumonia**				0.71
No	10 / 33	11 / 37	0.89 [0.37, 2.15]	
Yes	174 / 606	149 / 603	0.82 [0.66, 1.02]	
**Days between admission and randomization**				0.37
0	50 / 207	51 / 199	1.05 [0.71, 1.56]	
1 - 2	71 / 251	64 / 282	0.74 [0.53, 1.04]	
>=3	63 / 181	45 / 159	0.78 [0.53, 1.14]	

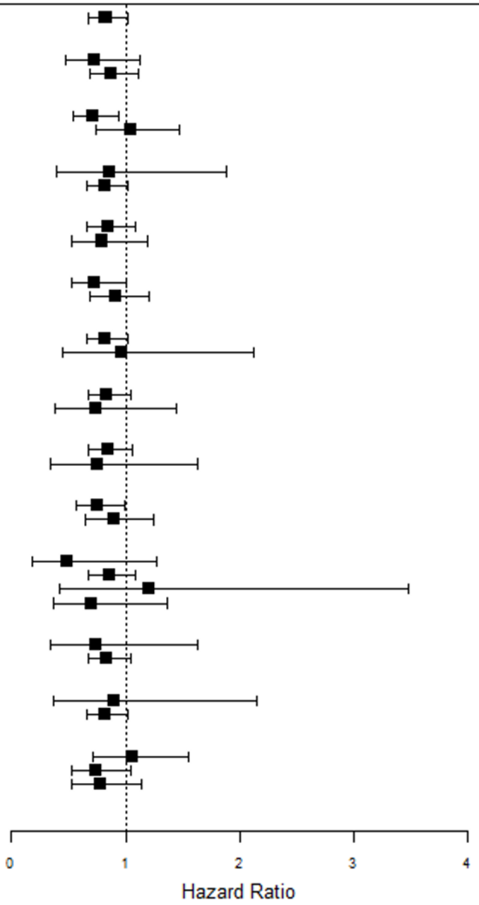

[a] p-value for interaction

© 2021 Diaz R et al. *JAMA Network Open.*

eFigure 2. Forest Plot for Primary and Secondary Outcomes Considering the Per Protocol Population

Outcome	Usual Care	Colchicine	HR [95% CI]
**Co-Primary - 28 days**			
Composite	181 / 634	117 / 515	0.75 [0.60, 0.95]
Mortality	140 / 634	98 / 515	0.83 [0.64, 1.07]
**Secondary - 28 days**			
New intubation or death from RF	171 / 634	105 / 515	0.72 [0.56, 0.92]
New intubation or death from N-RF	114 / 634	76 / 515	0.79 [0.59, 1.06]
Mortality from RF	124 / 634	83 / 515	0.79 [0.60, 1.04]
Mortality from N-RF	16 / 634	15 / 515	1.09 [0.54, 2.21]
**Secondary - In hospital**[a]			
Composite	177 / 634	113 / 515	0.79 [0.64, 0.97]
Mortality	136 / 634	94 / 515	0.85 [0.67, 1.08]

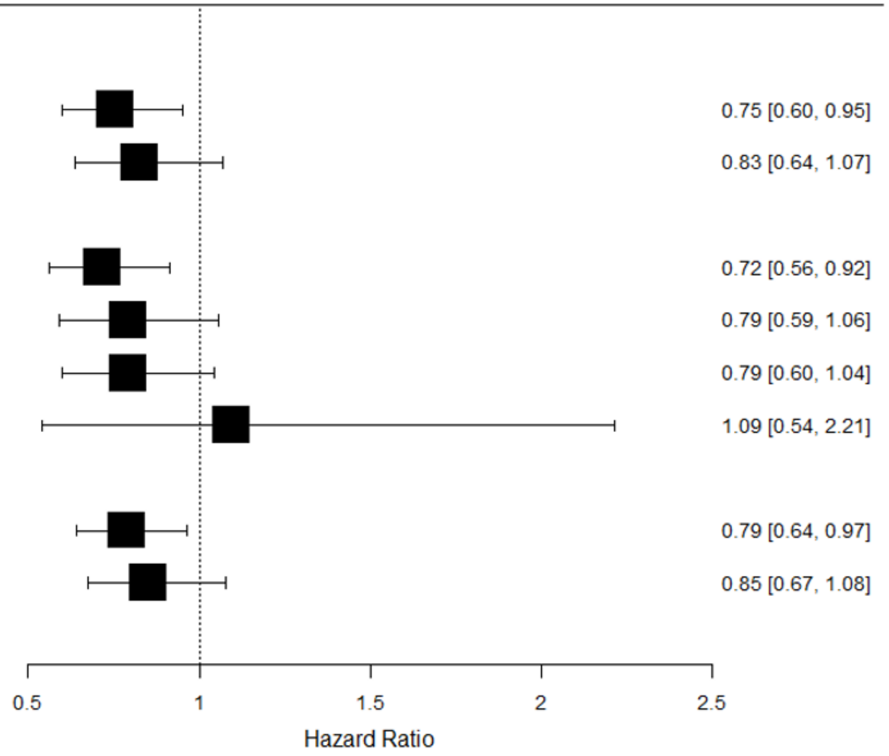

Patients are analyzed within the intervention group to which they were randomized after exclusion of patients who were non-compliant.
Patients defined as non-compliant for the colchicine group did not receive colchicine or received colchicine but discontinued (N=125).
Patients defined as non-compliant for the usual care group received colchicine (N=5).
Abbreviations: RF, respiratory failure; N-RF, Non respiratory failure
[a] Relative Risk and 95% CI for in-hospital outcomes

© 2021 Diaz R et al. *JAMA Network Open.*
