# Supplementary material for: Effect of Colchicine vs Usual Care Alone on Intubation and 28-Day Mortality in Patients Hospitalized With COVID-19: A Randomized Clinical Trial
Source: JAMA Netw Open. 2021 Dec 29;4(12):e2141328. doi: 10.1001/jamanetworkopen.2021.41328 (PMC8717104; doi:10.1001/jamanetworkopen.2021.41328)
Supplement: Supplement 3. — The ECLA PHRI COLCOVID Trial Investigators [file jamanetwopen-e2141328-s003.pdf]

Supplemental Online Content: Nonauthor Collaborators

*Indicates required information. Only first name, last name, and suffix will appear in PubMed.

*Group Name(s): The ECLA PHRI COLCOVID Trial Investigators

*First Name and Middle Initial(s)	*Last Name	*Suffix (eg, Jr, III)	Academic Degrees	Institution	Location (city, state/province, country)	Role or Contribution, eg, chair, principal investigator	Group (if more than 1 Group listed in the byline) and/or Subgroup (eg, Steering Committee)
Lorena	Lopez		MD	Centro Modelo de Cardiología	San Miguel de Tucumán, Tucu	Site Co-Investigator	
Juan	Muntaner		MD	Centro Modelo de Cardiología	San Miguel de Tucumán, Tucu	Site Principal Investigator	
Antonela	Bobato		MD	Clínica 25 de Mayo	Mar del Plata, Buenos Aires	Site Co-Investigator	
Gonzalo	Corral		MD	Clínica 25 de Mayo	Mar del Plata, Buenos Aires	Site Principal Investigator	
Gustavo	Benavent		MD	Clínica CEMEP	Rio Grande, Tierra del Fuego	Site Co-Investigator	
Diego	Espinel		MD	Clínica CEMEP	Rio Grande, Tierra del Fuego	Site Principal Investigator	
Sandra M.	Del Valle Almagro		MD	Clínica Fusavim Privada	Villa María, Córdoba	Site Co-Investigator	
Eleonora E.	Montenegro		MD	Clínica Fusavim Privada	Villa María, Córdoba	Site Principal Investigator	
Adrian	Núñez		MD	Clínica Monte Grande	Monte Grande, Buenos Aires	Site Co-Investigator	
Lisandro	Pérez Valega		MD	Clínica Monte Grande	Monte Grande, Buenos Aires	Site Principal Investigator	
Martin	Christin		MD	Clínica Olivos	Vicente Lopez, Buenos Aires	Site Co-Investigator	
Leda	Guzzi		MD	Clínica Olivos	Vicente Lopez, Buenos Aires	Site Principal Investigator	
Gabriela	Finelli		MD	Clínica Privada del Prado	Córdoba, Córdoba	Site Co-Investigator	
Lilina B.	Schiavi		MD	Clínica Privada del Prado	Córdoba, Córdoba	Site Principal Investigator	
Eduardo	Ferro Queirel		MD	CORDIS Instituto del Corazón	Resistencia, Chaco	Site Co-Investigator	
Luis M.	Moltrasio		MD	CORDIS Instituto del Corazón	Resistencia, Chaco	Site Principal Investigator	
Horacio A.	Fermín		MD	Hospital Británico	CABA, Buenos Aires	Site Co-Investigator	
Jorge V.	Martínez		MD	Hospital Británico	CABA, Buenos Aires	Site Principal Investigator	
Omar	Gutiérrez		MD	Hospital de Campaña de Ciudad Cultural	San Salvador de Jujuy , Jujuy	Site Principal Investigator	
Eleonora	Cunto		MD	Hospital de Infecciosas Francisco Javier M	CABA, Buenos Aires	Site Co-Investigator	
Pablo A.	Saúl		MD	Hospital de Infecciosas Francisco Javier M	CABA, Buenos Aires	Site Principal Investigator	
María del Pilar	Cabrera Maciel		MD	Hospital del Este Eva Perón	Banda del Río Salí, Tucumán	Site Principal Investigator	
Javier	Muntaner		MD	Hospital del Este Eva Perón	Banda del Río Salí, Tucumán	Site Co-Investigator	
Damián	Lerman		MD	Hospital Escuela Eva Perón	Granadero Baigorria, Santa Fe	Site Principal Investigator	
Paula I.	Truccolo		MD	Hospital Escuela Eva Perón	Granadero Baigorria, Santa Fe	Site Co-Investigator	
Adrián	Armano		MD	Hospital Interzonal Presidente Perón	Avellaneda, Buenos Aires	Site Co-Investigator	
Esther V.	Jalife		MD	Hospital Interzonal Presidente Perón	Avellaneda, Buenos Aires	Site Principal Investigator	
Romina M.	Bertuzzi		MD	Hospital Italiano	Córdoba, Córdoba	Site Principal Investigator	
María Inés	Jean Charles		MD	Hospital Italiano	Córdoba, Córdoba	Site Co-Investigator	

Supplemental Online Content: Nonauthor Collaborators

*Indicates required information. Only first name, last name, and suffix will appear in PubMed.

*First Name and Middle Initial(s)	*Last Name	*Suffix (eg, Jr, III)	Academic Degrees	Institution	Location (city, state/province, country)	Role or Contribution, eg, chair, principal investigator	Group (if more than 1 Group listed in the byline) and/or Subgroup (eg, Steering Committee)
Pablo A.	Fernandez		MD	Hospital Municipal	Chivilcoy, Buenos Aires	Site Principal Investigator	
Alberto	Caccavo		MD	Hospital Municipal "Dr. Raúl A. Caccavo"	Coronel Suárez , Buenos Aires	Site Co-Investigator	
Nicolás	Vittal		MD	Hospital Municipal "Dr. Raúl A. Caccavo"	Coronel Suárez , Buenos Aires	Site Principal Investigator	
Lucia	Lampone Tappata		MD	Hospital Municipal de Agudos Dr. Leónid	Bahía Blanca, Buenos Aires	Site Co-Investigator	
Diego M.	Murizzi		MD	Hospital Municipal de Agudos Dr. Leónid	Bahía Blanca, Buenos Aires	Site Principal Investigator	
Brenda L.	Fernandez		MD	Hospital Municipal Eva Perón	Merlo, Buenos Aires	Site Principal Investigator	
Antonio	Montes de Oca		MD	Hospital Municipal Eva Perón	Merlo, Buenos Aires	Site Co-Investigator	
Guadalupe	Diaz Vega		MD	Hospital Municipal Manuel B. Cabrera	Coronel Pringles, Buenos Aire	Site Co-Investigator	
Felipe N.	Queti		MD	Hospital Municipal Manuel B. Cabrera	Coronel Pringles, Buenos Aire	Site Principal Investigator	
Luis A.	Calafell		MD	Hospital Municipal Pigüé	Pigüé, Buenos Aires	Site Principal Investigator	
Mariano	Sequeira		MD	Hospital Municipal Pigüé	Pigüé, Buenos Aires	Site Co-Investigator	
Ricardo	León de la Fuente		MD	Hospital Papa Francisco	Salta, Salta	Site Principal Investigator	
Julio	Núñez Burgos		MD	Hospital Papa Francisco	Salta, Salta	Site Co-Investigator	
Sonia	Del Valle Armaraz		MD	Hospital Paterson	San Pedro, Jujuy	Site Co-Investigator	
Patricia	Flores		MD	Hospital Paterson	San Pedro, Jujuy	Site Principal Investigator	
Mariana	Bellanting		MD	Hospital Privado	Rosario, Santa Fe	Site Principal Investigator	
Narela	Blazevich		MD	Hospital Provincial	Rosario, Santa Fe	Site Principal Investigator	
Baltasar	Finucci Curi		MD	Hospital Provincial	Rosario, Santa Fe	Site Co-Investigator	
Romina P.	Cabrini		MD	Hospital Regional A Gutiérrez	Venado Tuerto, Santa Fe	Site Co-Investigator	
Martín E.	Langone		MD	Hospital Regional A Gutiérrez	Venado Tuerto, Santa Fe	Site Principal Investigator	
Álvaro E.	Figueroa		MD	Hospital Regional Río Gallegos	Río Gallegos, Santa Cruz	Site Co-Investigator	
Maria T.	Iglesias		MD	Hospital Regional Río Gallegos	Río Gallegos, Santa Cruz	Site Principal Investigator	
Maria Carolina	Alvero		MD	Hospital San Bernardo	Salta, Salta	Site Co-Investigator	
Cesar G.	Lemir		MD	Hospital San Bernardo	Salta, Salta	Site Principal Investigator	
José	Bonorino		MD	Hospital Universitario Austral	Derqui, Buenos Aires	Site Co-Investigator	
María Laura	Pereyra		MD	Hospital Universitario Austral	Derqui, Buenos Aires	Site Principal Investigator	
Ezequiel	Barral		MD	Instituto Investigaciones Clínicas Rosario	Rosario, Santa Fe	Site Co-Investigator	
Mariela	Rasmussen		MD	Instituto Investigaciones Clínicas Rosario	Rosario, Santa Fe	Site Principal Investigator	
María F.	Daglio		MD	Policlínico Central UOM	CABA, Buenos Aires	Site Co-Investigator	
Pablo A.	Saúl		MD	Policlínico Central UOM	CABA, Buenos Aires	Site Principal Investigator	
Mariano D.	Estofan		MD	Sanatorio 9 de julio	San Miguel de Tucumán, Tucu	Site Principal Investigator	

Supplemental Online Content: Nonauthor Collaborators

*Indicates required information. Only first name, last name, and suffix will appear in PubMed.

***First Name and Middle Initial(s)**	***Last Name**	***Suffix (eg, Jr, III)**	Academic Degrees	Institution	Location (city, state/province, country)	Role or Contribution, eg, chair, principal investigator	Group (if more than 1 Group listed in the byline) and/or Subgroup (eg, Steering Committee)
Francisco M.	Perea		MD	Sanatorio 9 de julio	San Miguel de Tucumán, Tucu	Site Co-Investigator	
Sebastián E.	Duhalde		MD	Sanatorio Argentino (Centro Médico Arg	San Miguel de Tucumán, Tucu	Site Principal Investigator	
Sandra M.	Del Valle Almagro		MD	Sanatorio de la Cañada	Villa María, Córdoba	Site Principal Investigator	
Eleonora E.	Montenegro		MD	Sanatorio de la Cañada	Villa María, Córdoba	Site Co-Investigator	
María Fernanda	Motta		MD	Sanatorio Güemes	CABA, Buenos Aires	Site Co-Investigator	
Ignacio	Romero		MD	Sanatorio Güemes	CABA, Buenos Aires	Site Principal Investigator	
Sebastián E.	Duhalde		MD	Sanatorio Integral Luz Medica SA	San Miguel de Tucumán, Tucu	Site Principal Investigator	
Guillermo	Isa Massa		MD	Sanatorio Integral Luz Medica SA	San Miguel de Tucumán, Tucu	Site Co-Investigator	
Celso F.	García		MD	Sanatorio Nuestra Señora del Rosario - E	San Nicolás, Buenos Aires	Site Principal Investigator	
Rubén	García Durán		MD	Sanatorio Nuestra Señora del Rosario - E	San Nicolás, Buenos Aires	Site Co-Investigator	
Elena	Cornejo Pucci		MD	Sanatorio Parque	Salta, Salta	Site Co-Investigator	
Silvia	Saavedra		MD	Sanatorio Parque	Salta, Salta	Site Principal Investigator	
Carolina	Bozikovich		MD	Sanatorio Parque	Rosario, Santa Fe	Site Co-Invetigator	
Luciano	Lovesio		MD	Sanatorio Parque	Rosario, Santa Fe	Site Principal Investigator	
María J.	Fernandez Moutin		MD	Sanatorio Plaza	Rosario, Santa Fe	Site Co-Investigator	
Cristian C. G.	Forciniti		MD	Sanatorio Plaza	Rosario, Santa Fe	Site Principal Investigator	
Hugo	Colombo		MD	Sanatorio Privado Duarte Quiros	Córdoba, Córdoba	Site Principal Investigator	
Nicolas	Sabas		MD	Sanatorio Privado Duarte Quiros	Córdoba, Córdoba	Site Co-Investigator	
Leonardo	Pilón		MD	Sanatorio y Clínica Lavalle	San Salvador de Jujuy , Jujuy	Site Co-Investigator	
Adriana P.	Steren		MD	Sanatorio y Clínica Lavalle	San Salvador de Jujuy , Jujuy	Site Principal Investigator	
