# Supplementary material for: Effect of Colchicine vs Usual Care Alone on Intubation and 28-Day Mortality in Patients Hospitalized With COVID-19: A Randomized Clinical Trial
Source: JAMA Netw Open. 2021 Dec 29;4(12):e2141328. doi: 10.1001/jamanetworkopen.2021.41328 (PMC8717104; doi:10.1001/jamanetworkopen.2021.41328)
Supplement: Supplement 4. — Data Sharing Statement [file jamanetwopen-e2141328-s004.pdf]

Data Sharing Statement

Diaz. Effect of Colchicine vs Usual Care Alone on Intubation and 28-Day Mortality in Patients Hospitalized With COVID-19. *JAMA Netw Open*. Published December 29, 2021. doi:10.1001/jamanetworkopen.2021.41328

Data
Data available: No

Additional Information
Explanation for why data not available: Please contact the corresponding author for any questions about data availability. The Executive Committee will review any proposals for novelty, feasibility, and scientific merit.
